# Supplementary material for: Exomes of Ductal Luminal Breast Cancer Patients from Southwest Colombia: Gene Mutational Profile and Related Expression Alterations
Source: Biomolecules. 2020 Apr 30;10(5):698. doi: 10.3390/biom10050698 (PMC7277822; doi:10.3390/biom10050698)
Supplement: Supplementary file 1 [file biomolecules-10-00698-s001.zip › Article-to-BIOMOLECULES_R1_v4Apr2020/CortesUrreaetal_R1_Figure_2.pdf]

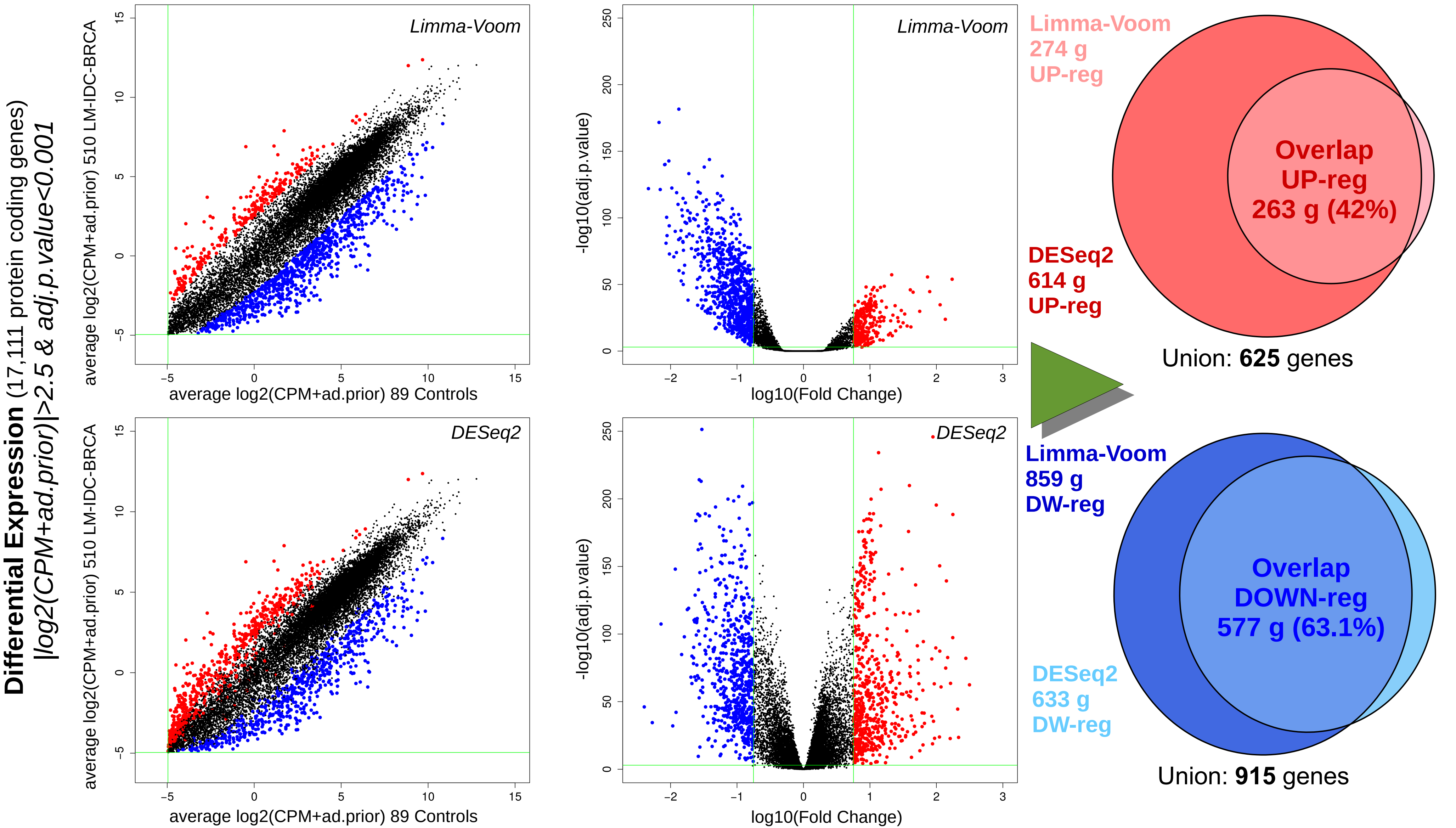

Differential Expression (17,111 protein coding genes)
|log2(CPM+ad.prior)|>2.5 & adj.p.value<0.001
Limma-Voom
average log2(CPM+ad.prior) 510 LM-IDC-BRCA
average log2(CPM+ad.prior) 89 Controls
Limma-Voom
-log10(adj.p.value)
log10(Fold Change)
DESeq2
average log2(CPM+ad.prior) 510 LM-IDC-BRCA
average log2(CPM+ad.prior) 89 Controls
DESeq2
-log10(adj.p.value)
log10(Fold Change)
Limma-Voom
274 g
UP-reg
DESeq2
614 g
UP-reg
Overlap
UP-reg
263 g (42%)
Union: 625 genes
Limma-Voom
859 g
DW-reg
DESeq2
633 g
DW-reg
Overlap
DOWN-reg
577 g (63.1%)
Union: 915 genes
