# Supplementary material for: Exomes of Ductal Luminal Breast Cancer Patients from Southwest Colombia: Gene Mutational Profile and Related Expression Alterations
Source: Biomolecules. 2020 Apr 30;10(5):698. doi: 10.3390/biom10050698 (PMC7277822; doi:10.3390/biom10050698)
Supplement: Supplementary file 1 [file biomolecules-10-00698-s001.zip › Article-to-BIOMOLECULES_R1_v4Apr2020/CortesUrreaetal_R1_Biomolecules707977_MANUSCRIPT.pdf]

biomolecules

MDPI

Article

Exomes of *Ductal Luminal* breast cancer patients from Southwest Colombia: gene mutational profile and related expression alterations

Carolina Cortes-Urrea [1,2]*, Fernando Bueno-Gutiérrez [1], Melissa Solarte [2], Miguel Guevara-Burbano [3], Fabian Tobar-Tosse [4], Patricia E. Vélez-Varela [5], Juan Carlos Bonilla [6], Guillermo Barreto [2], Jaime Velasco-Medina [7], Pedro A. Moreno [3] and Javier De Las Rivas [1]*

[1] Bioinformatics and Functional Genomics Group, Cancer Research Center (CiC-IMBCC, CSIC/USAL/IBSAL), Consejo Superior de Investigaciones Científicas (CSIC) and University of Salamanca (USAL), 37007 Salamanca, Spain. jrivas@usal.es
[2] Human Molecular Genetics Lab, Department of Biology, Universidad del Valle, Cali, Colombia.
[3] School of Systems Engineering and Computation, Universidad del Valle, Cali, Colombia.
[4] Department of Basic Health Sciences, Pontificia Universidad Javeriana Cali, Cali, Colombia.
[5] Department of Biology, University of Cauca, Popayán, Colombia.
[6] Imbanaco Medical Center, Cali, Colombia.
[7] School of Electrical and Electronic Engineering, Universidad del Valle, Cali, Colombia.
* Correspondence: jrivas@usal.es and karourrea64@gmail.com

Received: date; Accepted: date; Published: date

Abstract: Cancer is one of the leading causes of mortality worldwide. Breast cancer is the most frequent cancer in women, and in recent years it has become a serious public health problem in Colombia. The development of large-scale omic techniques allows simultaneous analysis of all active genes in tumor cells versus normal cells, providing new ways to discover the drivers of malignant transformations. Whole Exome Sequencing (WES) was obtained to provide a deep view of the mutational genomic profile in a set of cancer samples from Southwest Colombian women. WES was performed on 52 tumor samples from patients diagnosed with invasive breast cancer, which in most cases (33/52) were *Ductal Luminal* breast carcinomas (IDC-LM-BRCA). Global variant call was calculated and six different algorithms were applied to filter-out false positives and identify pathogenic variants. To compare and expand the somatic tumor variants found in the Colombian cohort, exome mutations and genome-wide expression alterations were detected in a larger set of tumor samples of the same breast cancer subtype from TCGA (that included DNA-seq and RNA-seq data). Genes with significant changes in both the mutational and expression profiles were identified, providing a set of genes and mutations associated with the etiology of *Ductal Luminal* breast cancer. This set included 19 single mutations identified as tumor driver mutations in 17 genes. Some of the genes (ATM, ERBB3, ESR1, TP53) are well-known cancer genes, while others (CBLB, PRPF8) presented driver mutations that had not been reported before. In the case of CBLB gene, several mutations were identified in TCGA IDC-LM-BRCA samples associated with overexpression of this gene and repression of tumor suppressive activity of TGF-Beta pathway.

Keywords: breast cancer; cancer genomics; genetic variant; whole exome sequencing; SNPs; differential expression; RNA-seq; bioinformatics; Limma-Voom; DESeq2.

1. Introduction

Among females, breast cancer is the most commonly diagnosed cancer and the leading cause of cancer death both in developed and developing regions [1]. Worldwide, 2.1 million female breast cancer cases were diagnosed in 2018, accounting for almost 1 in 4 cancer cases among women. The

disease is the most frequently diagnosed cancer in the vast majority of the countries (154 of 185) and is also the leading cause of cancer death in over 100 countries; the main exceptions are Australia/New Zealand, Northern Europe, Northern America (where it is preceded by lung cancer), and many countries in Sub-Saharan Africa (because of elevated cervical cancer rates) [2]. In Colombia, this disease is the second-most frequently diagnosed malignancy, representing the leading cause of death in women according to statistics from the National Cancer Institute (INC) from Colombia [3], that estimated in the period 2007-2011 around 7,600 new breast cancer cases diagnosed annually, with 2,226 annual breast cancer deaths [4].

Breast cancer is a heterogeneous complex of pathology, that includes multiple tumor subtypes with distinct biological features that lead to differences in response to treatment and in clinical outcome [5]. According to the *cellular classification*, the *Invasive Ductal Carcinoma* (IDC) is the most common subtype of breast cancer, accounting for about 80% of breast cancer diagnoses [6]. Moreover, considering the *molecular classification*, the luminal-like tumors (LM) are the most common subtypes among breast cancer [7]. Since cancer is a disease of complex genetic origin, it cannot be characterized from the study of a single gene or gene product. The genetic complexity inherent to cancer is primarily attributable to variation across patients, that suffer different somatically acquired alterations in different genes and present different rates of accumulation of such alterations [8]. In this scenario, the development of large-scale omic techniques, allowing the simultaneous analysis of all active genes in tumor cells versus normal cells, provides a new comprehensive way to discover the genetic alterations that can drive the expression and regulatory changes in the complexity of malignant transformations [9]. Currently in large genomic studies, such as The Cancer Genome Atlas (TCGA) project, DNA sequencing (DNA-seq) is the main technique utilized for mutation detection, either using a gene panel sequencing approach or whole exome sequencing approach; while RNA sequencing (RNA-seq) is performed to measure gene expression (looking for coding or non-coding genes) and transcript use (sometimes including splicing analyses to detect isoforms) [10].

In this work, we applied some of the omic technologies to the study of breast neoplasms. In particular, we used complete exome sequencing (Whole Exome Sequencing, WES) to provide a deep view of the mutational genomic profile in a set of cancer samples from Southwest Colombian women. Furthermore, we combined this information with the analysis of WES (DNA-seq) and genome-wide expression (RNA-seq) data from a subset of samples from the TCGA project with the same subtype of breast tumors of the Colombian cohort to infer the activation or alteration profile of genes and identify common pathogenic mutations. The integration of DNA-seq and RNA-seq data from the TCGA samples was also used to find Expression Quantitative Trait Loci (eQTLs), which allowed the identification of certain genomic loci that explained variation in expression levels of mRNAs based on allele modification. As a whole, the objective of this study was to find a set of gene-centered alterations identified as pathogenic somatic mutations in exomes from invasive breast carcinomas of *Ductal Luminal* subtype, in a cohort of patients of Southwest Colombia; and compare that set with a similar, but larger, cohort of TCGA patients. Pathogenic mutations were detected as somatic tumor variants corresponding to non-synonymous single nucleotide polymorphisms (nsSNPs). The results provide valuable information on the characteristics of this type of breast cancer and allow us to identify novel associations between genetic mutations and related genes in *Ductal Luminal* breast carcinomas. The work revealed well-known *cancer driver* genes, along with some new *cancer driver* mutations associated with the patient subpopulation we studied.

2. Materials and Methods

2.1. Ethical Approval

Tumor samples were collected from the volunteer participants with Informed Consent, following ethical guidelines approved by the University of Valle, the University of Cauca and the Imbanaco Medical Center; all based in Cali (Colombia).

2.2. Samples collection and DNA sequencing

A total of 52 breast cancer (BRCA) patients and 7 controls from Southwest Colombia were considered for this study. Samples were taken from breast tumor tissue in stages I to IV. No chemotherapy or radiotherapy had been applied to the patients before the collection of the tumor biopsies. The anatomopathological diagnosis of the breast cancer samples indicated that they were *Invasive Ductal Carcinomas* (IDC) (42/52 samples) and *Invasive Lobular Carcinomas* (ILC) (10/52 samples). DNA was extracted from the samples with Invitrogen PureLink Genomic DNA Mini Kit and sequenced by Macrogen Inc., using an Illumina HiSeq 4000 System at a 100x depth. Other 7 breast samples were collected from healthy tissue, to be used as controls in the study. The exomes of these control samples provided a large set of germline variants that were used to filter and clean out the exomes from the tumor samples to enrich them for somatic mutations.

2.3. Exome mapping and genetic variant calling

Sequencing data sets were mapped to the reference human genome (hg19/NCBI GRCh37) with BWA-MEM 0.7.8-r455, and Picard 1.115 was used to remove duplicates. Sequences were mapped using Seqmule 1.2.6 (locally adapted to run with the Slurm scheduler) and then a consensus of variants was obtained running HaplotypeCaller from GATK-lite 2.3-9, SAMtools 0.1.19 and FreeBayes 0.9.14 with default parameters.

In the analysis of the raw exome sequences, a coverage threshold of 100x was applied in order to have a clear mapping of each genomic locus and to identify well all the variants found. Statistical analysis and quality scores were computed for each single nucleotide variant, and variants with low scores were removed. The consensus of the three tools indicated above (GATK, SAMtools and FreeBayes) was taken into account for each variant, and if there was not complete agreement between them, after manual inspection, the variant was not considered. Variant annotation (looking for *non-synonymous single nucleotide polymorphisms*, nsSNPs) was performed with ANNOVAR. Data from 1000 Genomes, dbSNP, ExAC (Exome Aggregation Consortium) and specially data from COSMIC (Catalogue of Somatic Mutations in Cancer) were used in the annotation of variants. In this way, after all these steps, we found a first raw set of 60,026 variants (SNPs) in 14,634 genes. Variants present in any of the 7 exomes of the control breast tissue samples were discarded, producing a second set of 41,404 variants. See steps 1 to 4, in **Figure 1**, which presents the workflow that we used to select variants. In a third filter step, each of the 41,404 variants were evaluated for the damaging / pathogenic effect on the corresponding gene using 6 different tools: SIFT [11], PolyPhen-2 [12], MutationTaster 2 [13], FATHMM [14], CADD [15] and GERP++ [16]. Each of them was used with the following pathogenity thresholds: SIFT <0.05; PolyPhen-2 >=0.98; MutationTaster A or D; FATHMM D; CADD >=20; and GERP++ >=2. This provided a strict filtering that resulted in the identification of 1,079 pathogenic variants present in 845 genes.

2.4. Variant prioritization based on greater harmful impact on protein

In order to focus on the best predictions about the functional effects of the nsSNPs in the corresponding gene-products and their identification as somatic mutations, we applied some more stringent filters. Once the 1,079 variants were identified, the 10% less pathogenic variants for each of 4 quantitative tools (SIFT, PolyPhen-2, CADD and GERP++) were discarded (step 4 in **Figure 1**). In an alternative fourth step (step 4′ in **Figure 1**), we removed the 20% less pathogenic variants for each of the 4 quantitative pathogenity predictor methods (also starting from the 1,079 pathogenic variants). This last step provides only the consensus variants that were above the threshold for all four methods. This led to the identification of 508 highly pathogenic mutations finally selected, identified in the 52 exomes analysed and present in 432 genes. For each variant, we had the specific pathogenicity values provided by the tools, therefore, these 508 variants were ranked from more to less confident. This final set represents the most significant signal obtained.

2.5. Selection of samples from TCGA for comparative analysis with Colombian samples

We assessed the clinical characteristics of our cohort of Colombian patients to select a similar cohort of patients from TCGA and study them together. In this selection, we pursue a series of clinical

and phenotypic similarities between these two cohorts of patients to allow that the in-house Colombian WES data could be compared with WES and RNA-seq data from TCGA. As indicated above, in order to do this comparative analysis of genetic mutations (somatic variants) and expression data, it was very important to previously select specific cancer subtypes. Since most of the Colombian patients were *Ductal* and *Luminal* (33/52, 63.5%), our study focused on the analysis of this specific cancer subtype: *Invasive Ductal - Luminal - Breast Cancer* (IDC-LM-BRCA). Therefore, considering the characteristics of the Colombian samples we did a selection of a similar set of samples from TCGA. These similarities were the following:

(i) All patients from Colombia and from TCGA selection were women of similar age: presenting an average of 61.6 years old at diagnosis (standard deviation ± 12.6) for the Colombian cohort; and average of 57.3 years (SD ± 13.2) for the selected TCGA patients.
(ii) Both cohorts of patients were mostly White. A recent genetic study by Norris et al., 2017 [17] stated that the population from Antioquia, a close Colombian state culturally very similar to the patient's region (Valle del Cauca), shows averages of: 64% European ancestry, 29% Native American ancestry and 7% African ancestry. The majority of the selected TCGA patients were also White of European Ancestry (496/770, 64%). Therefore, to a large extent the Colombian and the TCGA patients have a similar genetic background. The remaining TCGA patients were: Black or African American (148/770, 19.2%), Asian (47/770, 6%), American Indian or Alaska Native (1/770, 0.01%), and not reported race (78/770, 10%).
(iii) With respect to the cellular subtypes, all the breast cancer patients selected from TCGA were *Invasive Ductal Carcinoma*. In this way, we matched with the main cellular subtype of the WES samples from Colombia: 42/52 (81%) *Invasive Ductal Carcinoma* (IDC).
(iv) With respect to the breast cancer intrinsic subtypes, the whole set of 770 tumor samples from TCGA were: *Luminal A* (339), *Luminal B* (171), *Basal* (165), *Her2* (73) and *Normal* (22). For the comparison with the Colombian cohort we only used the *Luminal* samples (339 + 171 = 510), because the majority of the Colombian samples (within the *Ductal*) were of *Luminal* subtype.
(v) Tumor stage: in both groups of patients, the majority of the samples corresponded to stage I and II tumors: 81% of the Colombian patients, and 76% of the patients selected from TCGA. Furthermore, none of the in-house patients from Colombia or TCGA patients had metastases.

2.6. Selection of samples from TCGA for expression calculation

We were able to obtain genome-wide expression data from TCGA for 859 breast samples that were downloaded from the GDC DataPortal (https://portal.gdc.cancer.gov): 770 breast tumor samples and 89 controls. The data corresponded to the expression signal measured by RNA-seq of 60,423 genes per sample, provided as normalized counts. The TCGA biolinks tool [18, 19] was used to retrieve the expression data and information about the samples. Before making a more specific selection within this cohort of breast samples, we performed a global analysis to obtain a comparative overview of the transcriptomic signal of the 859 samples (770 tumors + 89 controls). To do this, we applied a multidimensional scaling analysis (MDS) to the global gene expression data. MDS is a nonlinear dimensionality reduction method that allows visualize the level of similarity between samples in a data set. The results of this analysis, presented in **Supplementary Figure S1**, show the distribution of the samples within the two main dimensions, indicating a clear separation between the healthy breast samples (green dots in **Figure S1**) and the breast tumor samples. The analysis also reveals a clear separation between the *Luminal* and *Basal* breast cancer subtypes. Furthermore, in **Figure S1**, we appreciate a clear separation of *Luminal* from healthy *Controls* and a fair separation of *Luminal* from others (i.e. all other subtypes of breast tumors). This analysis supported the separation and specific selection of *Luminal* breast tumors for the main study in this work.

The RNA-seq expression counts from the 859 samples from TCGA were processed with the expression filter defined by Chen et al. (2016) [20], using the *filterByExpr* function of the algorithm *edgeR* (used as an R package). Then, a procedure was applied to recover 780 genes that were filtered out by *filterByExpr*, but that have significant expression in some specific subtype (as explained in the next section). In this way, the data from 27,603 genes (26,823 genes that passed *filterByExpr* + 780

recovered genes) were normalized with the *calcNormFactors* function from *edgeR*. This function uses the weighted trimmed mean of M-values method proposed by Robinson and Oshlack (2010) [21] to normalize the expression data and calculate CPM (counts per million).

2.7. Recovery of some genes expressed only in some groups

The *filterByExpr* function is applied to filter all genes or genetic entities that have very low expression levels in most samples of the different groups or subtypes compared. We considered that some of these genes may be relevant just for some groups, and therefore a protocol was developed to recover the fraction of genes with significant expression only in one or two of the groups considered (*Luminal*, *Others* and *Control*). As a recovery threshold, first we calculated for each gene (in 60,423 genes) the mean expression (in counts) across the 859 samples. Second, we calculated the median of the means expression distribution, also in raw counts. This median was 2.256 counts. Finally, we chose 3 times this median as the selected threshold for recovery. Accordingly, genes filtered by *filterByExpr* that had average expression counts > 6.77 in one or two of the groups were recovered. So, we found that 159 genes had average expression counts > 6.77 for the *Luminal* group and average counts < 6.77 in the other two groups (*Others* and *Control*); 224 genes had average counts > 6.77 only for the *Control* group; and 285 genes had average counts > 6.77 only for the *Others* group. Finally, we also found 22, 79 and 11 genes that had average counts < 6.77 only in one group: *Luminal*, *Control* or *Others*, respectively. These genes were also recovered since they had average expression counts > 6.77 in the other two groups. In total a set of 780 genes (159+224+285+22+79+11) were recovered to be included in the differential expression analysis.

2.8. Differential expression analysis of Ductal Luminal breast cancer (IDC-LM-BRCA) subtype

Differential expression analysis was carried on the normalized data with two independent methods: *Limma-Voom* [22] and *DESeq2* [23]; selecting the subtype of samples that were the objective of this work: *Ductal Luminal*. These samples (i.e., 510 from TCGA, 339 *Luminal A* plus 171 *Luminal B*), were compared with healthy *Controls* (89 samples from TCGA) and also with all the other subtypes of breast cancer (260 *Others*). Only the results of *Ductal Luminal* versus *Controls* were considered for the comparison with the variants derived from the exomes. The differential expression thresholds applied to select the most significant genes were: adjusted p.value < 0.001 and |log2FC| > 2.5 (i.e., absolute value of the log2 of the fold change). The p.values were adjusted by the Benjamini & Hochberg [24] procedure. A less stringent threshold (adjusted p.value < 0.05) was applied in order to find and annotate all the significant expression changes of the genes detected in the analysis of the exomes. In this way, we could combine expression data with genetic variants.

2.9. Functional analysis and annotation of the variants

In order to record the clinical and biological relevance of the genes found and their genetic alterations, obtained after prioritization, these alterations were analyzed with the CGI (Cancer Genome Interpreter) platform [25]. This systematizes the interpretation of cancer genomes, since it normalizes and automatizes the whole process. CGI identifies all the genomic alterations known as tumorigenic through the tool *OncodriveMUT* [26, 27], that includes the analysis of alterations of unknown clinical significance, and it uses all the available clinical evidence for the annotation of tumor variants that could act as biomarkers. CGI contains information of 5,314 validated mutations and 1,624 genomic biomarkers of response (sensitivity, resistance or toxicity) to 310 drugs in 130 cancer types. The selected genes were also mapped to 8 databases that include annotations for cancer genes and variants: CancerMine [28], UniProt (Universal Protein Repository, UniProt Consortium 2019) [29], COSMIC (Catalogue of Somatic Mutations in Cancer) [30], CIVic (Clinical Interpretation of Variants in Cancer) [31] , DoCM (Database of Cured Mutations in Cancer) [32], ClinVar (Clinically Relevant Variants) [33], OncoKB (Precision Knowledge Base in Oncology) [34] and NCG6.0 (Cancer Gene Network) [35]. A protein-protein interaction analysis was performed using STRING [36] and APID [37] for each of the prioritized candidates predicted as drivers mutations, in order to verify the interactions or associations between the selected genes. Finally, a functional enrichment analysis was performed using GeneTerm Linker [38] for the most significant differentially expressed genes.

2.10. Combined analysis of WES data from the Colombian and TCGA cohorts

To complement the approach illustrated in **Figure 1**, we used the WES data available for breast cancer patients from TCGA (https://www.cancer.gov/tcga) that were included in the expression analysis. Using this WES data from TCGA, we searched and identified mutations that were present also in the Colombian populations. The WES data (hg38/NCBI GRCh38) including somatic mutations of the primary tumors of 713 patients with *Invasive Ductal Carcinoma* from TCGA project were downloaded from GDC data portal. The data contained 79,508 different mutation sites. As indicated above, the data set was filtered to include only the 476 samples from the patients of *Ductal Luminal breast cancer* subtype (IDC-LM-BRCA) (476/713, 67%). Within these samples the number of mutations sites found was 43,213. Moreover, all these 476 patients were within the set of 510 *Ductal Luminal breast cancer* patients used in the differential expression analysis (**Figure 1**). Once defined the specific tumor subtype studied, we combined the Colombian and the TCGA data sets (i.e., 33 and 476 WES samples, respectively) to search for their common mutation sites. The WES data from Colombia were prepared as explained in sections 2.2 and 2.3. However, to achieve a better comparison of both WES data, the pathogenicity filters were not taken into account, since such pathogenic information was not available in the same way for the samples from TCGA. Therefore, we took the Colombian WES data prior to the application of the filters (which contained 45,454 mutation sites) and combined it with the 43,213 mutation sites found in the TCGA WES data, to find the intersection of both sets.

3. Results and Discussion

3.1. Analysis of the Whole Exome Sequencing data to identify relevant genetic variants

Figure 1 presents a workflow indicating the steps given to select the most relevant variants present in the WES data set of breast cancer samples studied. When the analysis was narrowed to the 33/52 *Ductal Luminal* patients (IDC-LM-BRCA), the set of variants was reduced to 339 in 304 genes.

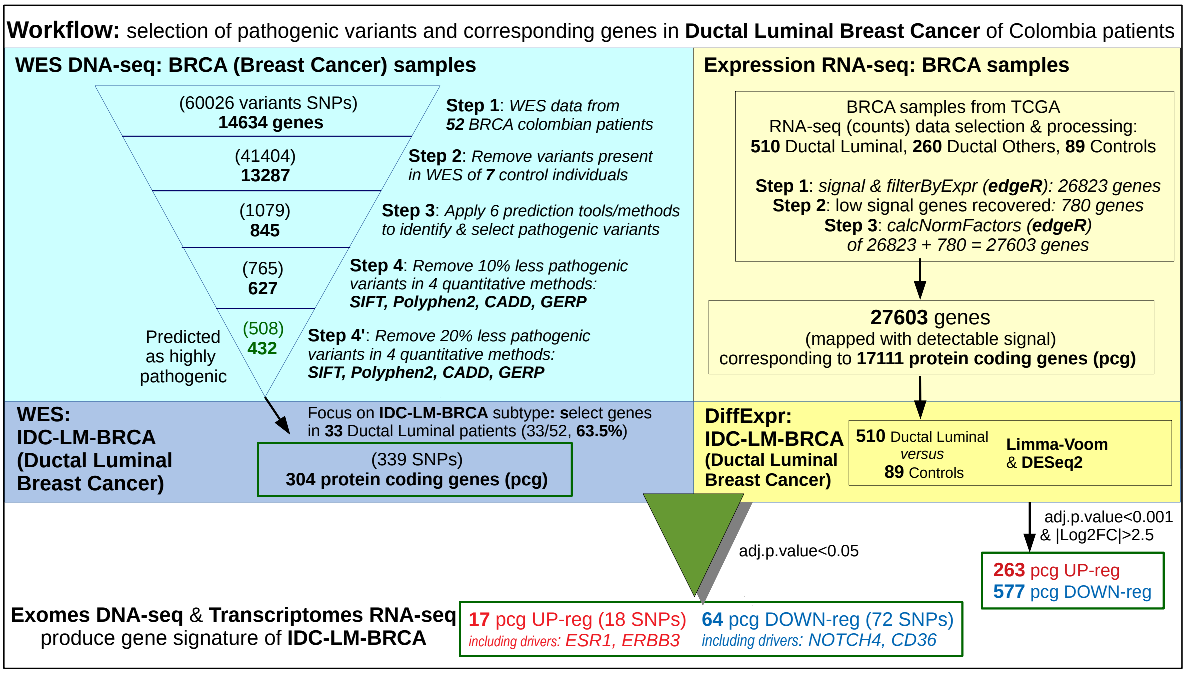

Figure 1. Workflow of the parallel analysis of WES DNA-seq data and Expression RNA-seq data from *Ductal Luminal* breast cancer samples, done to select pathogenic variants and corresponding altered genes in a set of Southwest Colombian patients.

The results of the differential expression obtained in the comparison of 510 samples of *Ductal Luminal* versus 89 *Controls* were checked to determine which of the genes with variants may suffer a concomitant expression alteration. Despite the fact that the experiments were done with different

samples, we found that 81 of the 304 genes (26.44%) showed differential expression for *Luminal* vs *Control* (adj.p.value < 0.05 for Limma-Voom & DESeq2). In addition, 17 of these genes were upregulated and 64 downregulated, indicating enrichment in the suppression signal. Some relevant genes that included variants and were found in the differential expression analysis were: ESR1 and ERBB3 overexpressed; NOTCH4 and CD36 repressed. The identification of the estrogen receptor (ESR1) as a genetically activated and mutated gene in patients with *Ductal Luminal* breast cancer is very consistent with the fact that ESR1 is a well-known marker and driver of *Luminal* breast cancer.

Of the highly pathogenic SNPs found in genes that showed differential expression (i.e., 18 SNPs present in upregulated genes and 72 SNPs present in downregulated genes, **Figure 1**); 19 SNP variants were identified as *driver mutations* by the Cancer Genome Interpreter (4 known, 13 reported and 2 new). Therefore, 2 SNP variants considered as somatic mutations in the tumors had never been reported. These 19 driver mutations are listed in **Table 1**.

The differential frequency distribution of genetic variants detected in our study shared with other populations of the world showed different overlaps: 26.7% with European (non-Finnish) population, 20% with Latino population and 13% with African population. This reflects a high level of ethnic background miscegenation of the Colombian population. In fact, in the whole country around 20% of Colombians can be identified with African ancestry, which shows the second largest population of Afro-descendants in continental Latin America. However, these proportions change quite a lot in different regions. For example, the Chocó region shows mostly African ancestry (76%) with an almost uniform division among European fractions (13%) and Native Americans (11%). By contrast, the Medellin region has mainly European ancestry (75%), followed by Native Americans (18%) and Africans (7%) [39]. The Colombian population of Valle del Cauca, which is the region of the patients in this study, shows genetic characteristics very similar to the population of Antioquia in Colombia, which in a recent genetic study showed around 65% European ancestry, around 30% Native American ancestry and 5-9% African ancestry [17]. The majority of the selected patients of the TCGA cohort used in our study were also white of European ancestry (64%).

The exomes studied here initially provided a large set of 60,026 SNP variants. As indicated in **Figure 1** and described in Materials and Methods, several consecutive steps were applied to identify and select pathogenic mutations that reach a set of 508 alterations, verified using four different quantitative methods. These 508 alterations were furtherly filtered to include only the ones present in the *Ductal Luminal* patients (IDC-LM-BRCA subtype), reaching the number of 339 SNP alterations included in 304 protein coding genes (pcg) (**Figure 1**). The complete list of these 339 variants, including complete details on the location of the mutation (aa position in the protein), as well as the patients who have each alteration, is provided as **Supplementary Table S2**. The variants were classified, using the Cancer Genome Interpreter, into: (i) population *polymorphisms* (that are population variants no currently related to cancer), and somatic mutations due to cancer of two types: (ii) *transient mutations* predicted as passenger; and (iii) *driver mutations* identified as causal in cancer (that can be - known, reported or new). In this analytical separation of mutations, priority was given to sequence alterations whose prediction corresponded to conductive cancer mutations (i.e., to driver mutations). As indicated above, **Table 1** includes the SNP variants that were identified as driver mutations: 17 known or already reported, plus 2 newly reported in this work.

3.2. Genes including genetic variants considered driver mutations

Among the 17 selected genes with at least one driver mutation, some canonical cancer genes were found, such as: ATM (Serine / Threonine Kinase ATM), ERBB3 (Erb-B2 Tyrosine Kinase 3 Receptor), MLH1 (Mismatch Repair Protein Mlh1 DNA), ESR1 (Estrogen Receptor 1), NOTCH1 (Notch Receiver 1) and TP53 (Tumor Protein P53). These are involved in fundamental pathways and processes of carcinogenesis, such as: the MAPK and PI3K-AKT signaling pathway (TP53 and ERBB3), estrogen signaling pathway (ESR1), apoptosis, cell death and cell growth (ATM and TP53).

Table 1. Genes and associated exonic variants, presented as driver mutations (known or predicted) of breast cancer in women from Southwest Colombia.

Gene HGNC Symbol	Nucleotide Change	Protein AA Change	dbSNP_ID(rs)	Frequency in IDC-LM-BRCA	Cancer-Genome Interpreter prediction	SNPs (known, reported, new)	Human population with more frequency
**ABCB4**	c.G2363A	p.R788Q	rs8187801	**3/33**	Driver_mutation	reported	ExAC_AFR
**ATM**	c.C7375G	p.R2459G	rs730881383	**1/33**	Driver_mutation	reported	ExAC_OTH
**ATM**	c.C7468T	p.L2490F	rs753262623	**1/33**	Driver_mutation	reported	ExAC_SAS *
**CD36**	c.G1016T	p.G339V	rs146027667	**1/33**	Driver_mutation	**known**	ExAC_OTH
**CHD8**	c.C871T	p.L291F	rs192989929	**1/33**	Driver_mutation	reported	ExAC_OTH/ExAC_AMR
**DPYD**	c.A2846T	p.D949V	rs67376798	**1/33**	known in cancer	reported	ExAC_NFE
**EPHA1**	c.C2371T	p.R791C	rs766301333	**1/33**	Driver_mutation	reported	ExAC_NFE
**ERBB3**	c.G2167C	p.V723L	rs189789018	**1/33**	Driver_mutation	**known**	ExAC_AMR
**ESR1**	c.G1138C	**p.E380Q**[#]	rs1057519827	**1/33**	Driver_mutation	**known**	all populations similar
**MLH1**	c.A1129G	p.K377E	rs35001569	**1/33**	Driver_mutation	reported	ExAC_NFE
**MSH3**	c.T2732G	p.L911W	rs41545019	**2/33**	Driver_mutation	reported	ExAC_NFE
**NOTCH1**	c.G2983A	**p.G995S**[##]	rs868369610	**1/33**	Driver_mutation	reported	all populations similar
**NOTCH4**	c.G2504T	p.G835V	rs9267835	**2/33**	Driver_mutation	**known**	ExAC_AFR/ExAC_AMR
**STAT6**	c.C1069T	p.R357W	rs776930978	**1/33**	Driver_mutation	reported	all populations similar
**TP53**	c.G338T	p.G113V	rs121912656	**1/33**	Driver_mutation	reported	ExAC_EAS
**TP53**	c.T215A	p.L72Q	rs1057519997	**1/33**	Driver_mutation	reported	all populations similar
**UPF3B**	c.G1082A	p.R361H	rs143538947	**1/33**	Driver_mutation	reported	ExAC_AFR
**CBLB**	c.G1972A	p.G658S	locus (chr:3q13.11;exon:13)	**1/33**	Driver_mutation	**new**	*NA*
**PRPF8**	c.G4153T	p.V1385F	locus (chr:17p13.3;exon:25)	**1/33**	Driver_mutation	**new**	*NA*

Populations are represented in the EXAC data. **AFR**: African/American, **AMR**: Latino, **EAS**: East Asian, **FIN**: Finish, **NFE**: Non-Finnish European, **SAS**: South Asian, **OTH**: Other.

ESR1 protein E380Q: This mutation is currently used as biomarker in BRCA

NOTCH1 protein G995S: This mutation is currently used as biomarker in BRCA

Additionally, some genes were found that had rarely been reported as altered in cancer studies, like: UPF3B (regulator of nonsense mediated mRNA decay) that encodes a protein that is part of a post-splicing multiprotein complex involved in both mRNA nuclear export and mRNA surveillance; and DPDY (dihydropyrimidine dehydrogenase) enzyme involved in the breakdown of nucleotides pyrimidines (uracil and thymine) when they are not needed. Finally, as shown in **Table 1**, we found two new driver mutations in two genes that have already been associated with breast cancer: PRPF8 and CBLB. PRPF8 (pre-mRNA processing factor 8) is a component of both U2- and U12-dependent spliceosomes, found to be essential for the catalytic step II in pre-mRNA splicing process. PRPF8 is a cancer related gene with different effect in different tissues and it may affect how RNA binding proteins mediate cancer-specific phenotypes [40]. CBLB (Cbl proto-oncogene B) encodes an E3 ubiquitin-protein ligase which promotes proteosome-mediated protein degradation by transferring ubiquitin from an E2 ubiquitin-conjugating enzyme to a substrate. It also functions as a negative regulator of T-cell activation. The CBLB gene can block the TGF-Beta pathway and has been associated with breast cancer [41]. In our study, we investigated the relationship between CBLB and the TGF-Beta pathway by analyzing the mutations and the expression levels found for this gene. This is explained in section 3.7.

3.3. Functional involvement in cancer of genes found with driver mutations

UPF3B encodes a protein that is part of a post-splicing multiproteic complex involved both in nuclear mRNA export and mRNA control, detecting mRNA with a defective reading frame and initiating nonsense-mediated mRNA decay (NMD). UPF3B has been linked to cancer because some tumor cells use NMD to destroy mRNAs from key tumor supressor genes [42]. This is the case, for example, in breast and ovarian cancer where the nonsense-mediated mRNA decay pathway triggers degradation of most BRCA1 mRNAs [43]. Another gene eventually linked to cancer is DPDY. As indicated above, the protein encoded by DPDY is a catabolic enzyme of pyrimidines (dihydropyrimidine dehydrogenase) that participates in the first step of the decomposition of pyrimidines, converting uracil into another molecule called 5,6-dihydrouracil and thymine into 5, 6-dihydrotothiamine. The molecules generated from this process can be used in other cellular processes. Cancer cells exhibit a very active and dynamic metabolic control to have sufficient supply of nucleotides and other macromolecules to grow and proliferate. In fact, cancer cells tune the signaling pathways to empower *de novo* synthesis of nucleotides. This enables that the metabolic requirements for cell growth are satisfied and the synthesis of nucleic acids and proteins can take place [44]. Alterations in the DPDY gene could alter the normal functioning of the enzyme encoded by this gene, contributing to the proliferation of cancer cells. Similarly, other defects in pyrimidine metabolism can increase the toxicity risk in cancer patients that received chemotherapy with drug 5-fluorouracil (5-FU), that is a pyrimidine analog.

The alteration in DPDY was identified in a patient positive for estrogen receptor (ER), progesterone receptor (PR) and HER2 receptor (triple positive) (**Supplementary Table S2**). This finding is very consistent with the literature, since there are reports of several studies related to the low response of ER+ tumors to conventional chemotherapy. In fact, patients with ER- tumors have more complete pathological responses to neoadjuvant chemotherapy than ER+ [45]. Luminal tumors have only a 6% complete pathological response to preoperative chemotherapy based on paclitaxel followed by 5-fluorouracil, doxorubicin and cyclophosphamide, contrary to 45% complete pathological response in the basal (ER-PR-) and HER2+ subtypes [46]. The same studies confirm that the luminal B subtype has even a worse response than luminal A. In this context, we can consider that the negative response to chemotherapy in the evaluated cases, can be related to the alteration in DPDY. As indicated above, PRPF8 is a central RNA splicing factor, essential for catalytic passage II in the splicing process prior to mRNA [47]. The disruption of RNA splicing causes genome instability and the factors involved in this process have been related to tumor suppression. Recurrent mutations in the PRPF8 gene have been observed in malignant myeloid tumors and are correlated with an increased proliferative capacity [48].

Two of the alterations predicted as cancer drivers in the *Ductal Luminal* subtype are well known breast cancer biomarkers: ESR1 mutation (E380Q) and NOTCH1 mutation (G995S) (**Table 1**). Biomarkers have many potential applications in oncology, including risk assessment, screening, differential diagnosis, prognosis determination, prediction of treatment response and disease progression monitoring [49]. Therefore, confirmation of specific biomarkers will have a very positive impact on the management of disease for patients with specific cancers. With regard to the effect of these mutations in pharmacological treatments, ESR1 mutation (E380Q) is sensitive to Fluvestran (hormone therapy) [50] and resistant to Tamoxifen (hormone therapy) [51]; and NOTCH1 mutation (G995S) is sensitive to the gamma-secretase inhibitors (GSI) that block NOTCH signaling [52].

3.4. Global differential expression of Ductal Luminal breast cancer samples

Differential expression analyses were carried with the *Limma-Voom* [22] and *DESeq2* [23] methods, as described in Materials and Methods, using RNA-seq data from TCGA, comparing 510 *Ductal Luminal* samples (339 *Luminal A* and 171 *Luminal B*) with 89 healthy *Controls*. The differential expression thresholds applied to select the most significant genes obtained with these two methods were: adjusted p.value < 0.001 and |log2FC| > 2.5. The genes that were significantly differentially expressed with both methods were selected. In this way, a significant set of 840 genes was identified, including 263 overexpressed genes and 577 repressed genes. The complete list of these genes, with their description, the corresponding p.values and the fold-change given by each method, are provided as **Supplementary Table S3**.

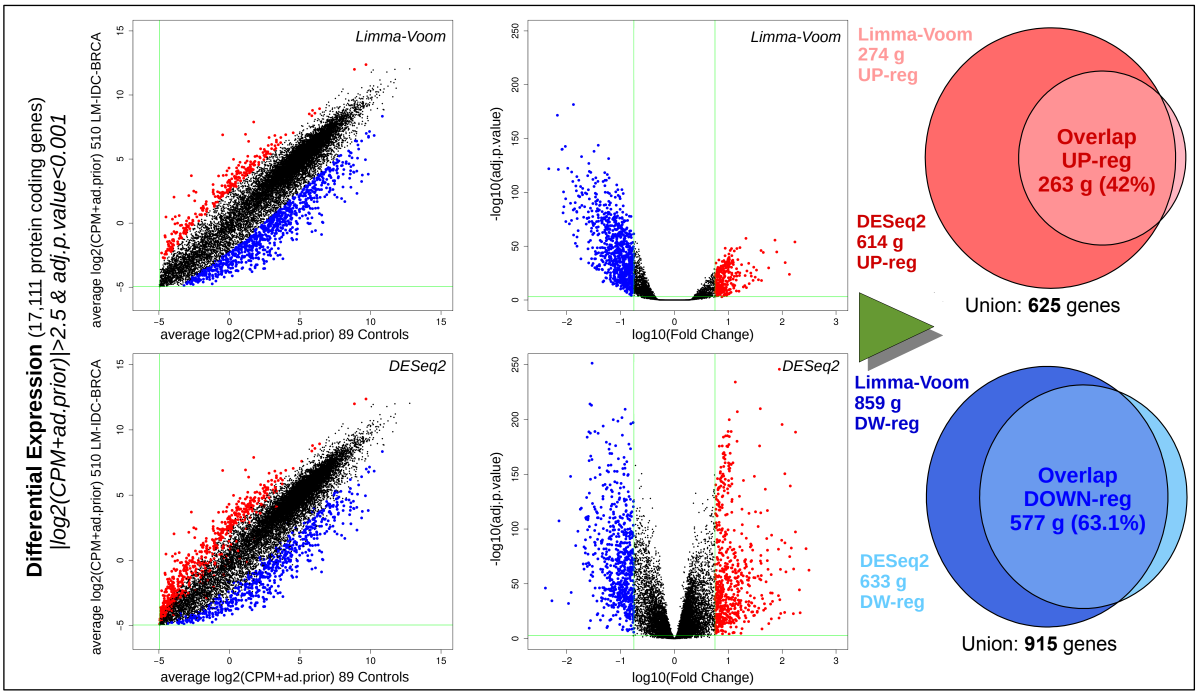

Figure 2. Scatter plots, volcano plots and proportional Venn diagrams showing the results of the differential expression analysis of RNA-seq data from 510 *Ductal Luminal* breast cancer samples versus 89 healthy *Control* samples. As indicated in the article, the analyses were done using two algorithms: *Limma-Voom* (upper scatter plot and volcano plot) and *DESeq2* (lower scatter plot and volcano plot). Upregulated genes are marked in red and downregulated genes are marked in blue.

As indicated above, the complete list of 840 genes corresponding to the overlapping differential expression signature (shown in **Figure 2**) is provided in **Supplementary Table S3**. This list includes up-regulated genes that are clear cancer markers, like the aurora kinases A and B (AURKA and AURKB), that are frequently amplified and overexpressed in cancer, and they are related to proliferation; the same as gene Ki-67 (MKI67). Other up-regulated genes are CEACAM5 and CEACAM6, associated with carcinogenesis; and many genes involved in the stimulation of mitosis

and the cell cycle: CCNB2 (cyclin B2), CDK1, CDC6, CDC20, CDC20B, CDC25C. One of the most altered pathways, according to a functional enrichment assignment in KEGG database, is *transcriptional misregulation in cancer* (i.e., KEGG pathway hsa05202) that includes genes like WT1 and MMP9, as well as several other matrix metallopeptidases highly overexpressed: MMP11 and MMP13. As a whole, we obtained a large gene differential expression signature characteristic of *Ductal Luminal* breast cancer samples derived from TCGA, even using fairly strict statistical thresholds and considering only the results of the superposition of two methods. In the next section, we looked for any gene that had significant differential expression in the *Ductal Luminal* breast cancer samples, and also that showed some alteration or mutation in the exome sequencing data.

3.5. Differential expression of Ductal Luminal breast cancer samples in genes that suffer mutations

The differential expression results corresponding to the comparison of 510 *Ductal Luminal* versus 89 *Controls* (i.e. the same samples as in the previous section), using a threshold of adjusted.p.value < 0.05 were crossed with the genes identified after all the WES data analyses (i.e., the 304 protein coding genes found for *Ductal Luminal* breast cancer). With this approach, a set of 81 genes were identified. The complete list of the 304 protein coding genes that include variants, combined with the differential expression data obtained for 81 genes derived from the comparison of *Ductal Luminal* versus *Controls*, are provided in **Supplementary Table S4**.

Figure 3 shows the chromosome location (in the X axis) of these 81 genes, together with their differential expression significance (in the Y axis) measured as -log10(adjusted.p.value) (taking the data from the values calculated with *DESeq2*). The concentration of these 81 genes along the genome was highest in chromosomes 6, 7 and 15. The genes marked as red dots in **Figure 3** were up-regulated in the RNA-seq data analysis of cancer samples with respect to healthy controls, and the genes marked as blue dots in **Figure 3** were down-regulated in cancer samples with respect to healthy controls. The four genes that were identified to have known driver mutations, according to our analysis of WES, are marked in this plot with a green box (**Figure 3**). These genes were also identified in the differential expression analysis: NOTCH4 and CD36 found repressed in the RNA-seq data; and ESR1 and ERBB3 found overexpressed in the RNA-seq data.

The identification of the estrogen receptor (ESR1) as an overexpressed gene, as well as a mutated gene in the data of our patients with *Ductal Luminal* breast cancer is worth noting, since it confirms and provides validation to the methodological approach of this study. ESR1 is a well-known positive biomarker of luminal breast cancer. Furthermore, several studies suggest that alteration of the estrogen receptor alpha gene (ESR1) can contribute to therapeutic resistance and metastasis in breast cancer [53, 54].

ERBB3 is a member of the human epidermal growth factor receptor (EGFR) family. ERBB3 is an important molecule in estrogen receptor positive breast cancers (ER+), which represents approximately 80% of all breast cancers. A high expression of this gene has been detected in ER+ and *Luminal* tumors [55]. Furthermore, elevated levels of ERBB3 correlate with progression of several solid tumors [56, 57]. In these reports, it has also been observed that ERBB3 mutations can activate MAPK and HER signaling in ER+ breast cancer cells. Additionally, ERBB3 activates the PI3K pathway, through its association with ERBB2 (HER2). In many cases, the effectiveness of hormonal therapy is nullified by the PI3K pathway, that remains very active along with high levels of ERBB2. This cancellation of hormonal therapy results in the activation of transcription factors that disrupts the epithelial polarity and leads to hyper-proliferation [47].

NOTCH4, a member of the NOTCH signaling pathway and the NOTCH family, plays an important role in cell development pathways, including proliferation, differentiation and apoptosis. NOTCH4 expression is inversely associated with the estrogen receptor (ER) and / or progesterone (PR) receptor, and is positively associated with a large tumor size, lymph node involvement and a more advanced stage of tumor lymph node metastasis. Its overexpression is more related to basal molecular subtypes. Thus, it is reasonable that NOTCH4 is down-regulated in the *Luminal* subtype.

The identification of the CD36 repressed gene in this study is consistent with the finding by Sun et al. 2018 [58], who reported that the repression of the CD36 gene in lung tumor samples inhibits cell proliferation, blocks the cell cycle in the G0 / G1 phase and inhibits cell migration.

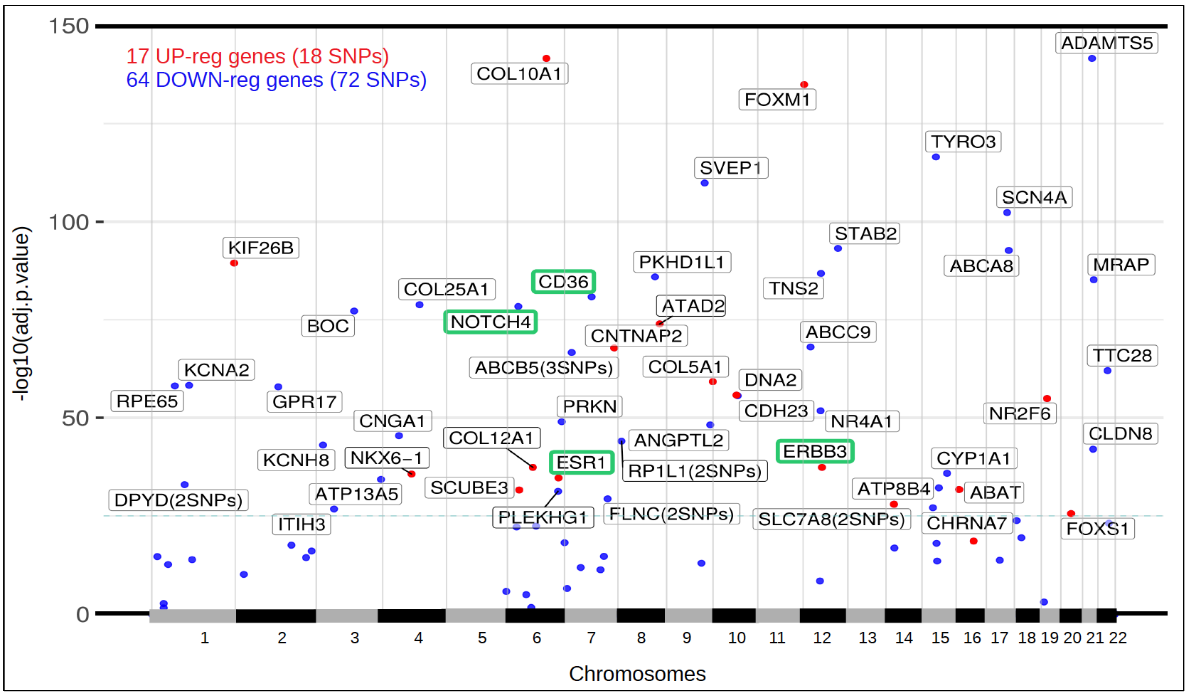

Figure 3. Plot of the chromosomal distribution (location) and differential expression (significance) of 81 genes that presented highly pathogenic mutations and expression alterations in *Ductal Luminal* breast cancer (IDC-LM-BRCA) patients.

3.6. Functional view of the genes altered in Ductal Luminal breast cancer

A functional analysis carried with Gene Term Linker [36] for the 81 deferentially expressed genes with highly pathogenic mutations in IDC-LM-BRCA showed a significant enrichment in processes associated with carcinogenesis and tumor progression, indicating that this set of genes constitutes a significant molecular signature of the malignant state of the samples studied.

In particular, genes DLL1, FOXS1, GJB5, KRT15, LAMA1, LAMA3, NOTCH4 and TGM5 were all downregulated and participate in the enrichment of the NOCTH4 signaling pathway (GO: 0007219) and cell adhesion regulation (GO: 0030155). Genes CD36, COL4A4, COL5A1, CTSG, FBN3, FLNC, LAMA1 and LAMA3 participate in the signaling pathway WNT, PI3K-AKT, calcium and MAPK. The MAPK pathway is the most frequently mutated signaling pathway in human cancer, currently considered a promising target for cancer therapy. This pathway plays a central role in the induction of responses such as cell proliferation, differentiation, growth, migration and apoptosis [59]. This pathway is initiated by an extracellular mitogenic stimulus that leads to the activation of RTK or GPCR. The MAPK / ERK pathway leads to phosphorylation and subsequent translocation of ERK in the nucleus. ERK activation plays a central role in the induction of cell cycle input and the suppression of negative regulators of the cell cycle [60]. The PI3K-AKT signaling pathway also regulates many normal cellular processes, including cell proliferation, survival, growth and motility. These processes are critical for tumorigenesis and the role of this pathway in oncogenesis has been extensively investigated. Many components of this pathway have been related to human cancer in studies that analyse both mutation and expression changes [61].

The identification of genes enriched in the WNT and calcium signaling pathways in the analysis of *Ductal Luminal* samples is an expected result, since both these pathways have been directly related to the tissue and the subtype evaluated in our work. The WNT signaling pathway is important for the development and remodeling of the breasts during pregnancy and lactation, and modifications

in the components have an impact on oncogenic transformation. Likewise, alterations in calcium homeostasis occur frequently in some pathological conditions such as malignant proliferation, and the entry of Ca has a decisive part in determining the concentration of Ca in the epithelial breast cells. Glandular breast proliferation, differentiation and lactation are regulated by several local and systemic hormones, of which estrogen is one of the most important ones. Estrogen regulators and their receptor are modulators of proliferation and differentiation of breast epithelial cells [62]. The effect of estrogen on the epithelial breast cells is mainly done through genomic regulation, but non-genomic mechanisms depend particularly on Ca signaling [63].

Another group of genes found in our set (constituted by ABCA13, ABCA8, ABCB5, ABCC9, ATAD2, ATP13A5, CFTR and DNA2), was enriched in ABC transporters and ATPase activity coupled to transmembrane movement of substances. The expression of these proteins is related to drug resistance and is an important obstacle for successful chemotherapy. Genes CFTR, CHRNA7, CLCNKB, CNGA1, KCNA2, KCNH8, SCN4A, SCN7A and SLC26A4 were associated with ion channels activated by voltage (GO: 0005244). In breast cancer, different types of ion channels other than Ca have been associated with tumorigenesis. Recently, voltage-dependent Na channels (VGSC) have been implicated in processes that lead to increased tumor aggressiveness [64]. This may be due to the fact that alteration in the proteins involved in the cell processes described can also contribute significantly to cellular mitotic biochemical signaling, cell cycle progression and cell volume regulation [65].

3.7. Mutations found in CBLB, a gene that inhibits the TGF-Beta pathway

The CBLB gene was one of the two genes for which we identified novel mutations in the Colombian cohort. CBLB and its paralog CBL are called proto-oncogenes and encode E3 ubiquitin-protein ligases. These genes are known to block the TGF-Beta pathway. Indeed, it has been reported that CBL genes enhance breast tumor formation by inhibiting tumor suppressive activity of TGF-Beta signaling pathway [41]. In our analysis of the 476 IDC-LM-BRCA samples from TCGA, the CBL and CBLB genes showed no significant change in expression compared to normal controls. However, two of the genes of the TGF-Beta pathway (TGF-Beta receptors TGFBR2 and TGFBR3) presented a very significant down-regulation in expression (adj.p.value < 0.001 with Limma-Voom and DESEq2), which indicated a probable inhibition of the TGF-Beta signaling pathway in the *Ductal Luminal* breast carcinomas. Furthermore, analysis of the whole exomes of the 476 IDC-LM-BRCA samples from the TCGA project revealed 13 distinct mutations in the CBLB gene, 12 of which had been reported as confirmed somatic tumor mutations (in COSMIC v90), and 6 of them corresponding to missense variants that are likely to damage the protein (**Supplementary Table S5**). Based on these exomes, we also evaluated whether there was any relationship between the mutations and the gene expression of CBLB. Thus, we found in our TCGA set of IDC-LM-BRCA samples, 6 patients with one or more of the 13 CBLB reported mutations. Comparison of the expression of CBLB in these mutated patients with the average expression in the non-mutated patients detected an overexpression of CBLB (adj.p.value = 0.0343) and a repression of a TGF-Beta receptor called TGFBR3L (adj.p.value = 0.0613). Therefore, these 13 mutations may be contributing to a more acute blockage of the TGF-Beta pathway in this group of breast cancer patients. A table with information on these 13 mutations is provided in **Supplementary Table S5**.

3.8. Common mutated genes in Ductal Luminal breast cancer patients from Colombia and TCGA

As described in section 2.10. (*Combined analysis of WES data from the Colombian and TCGA cohorts*) we performed the intersection of 43,213 mutation sites found in the 476 TCGA *Ductal Luminal* patients and the 45,454 mutation sites in the 33 Colombia *Ductal Luminal* patients. This combined analysis of WES samples from Colombia and TCGA provided a set of 29 common genes that were found mutated in both cohorts of *Ductal Luminal* breast carcinoma patients. These genes include 35 single nucleotide mutations, with the following 3 genes exhibiting multiple mutations: PIK3CA with 4 mutations, TP53 with 3, and MUC4 with 2. PIK3CA mutations represent one of the most common genetic aberrations in breast cancer. They have been reported to be present in over one-third of cases, with enrichment

in the *luminal* subtypes. The tumor suppressor gene TP53 is the most frequently mutated gene in somatic cells of human cancers. All the information on these mutations is included in an additional supplementary table called: **Supplementary Table S6**. Along with the genes that overlap between the Colombian and TCGA data sets, we also looked for the specific mutations that matched between this list of 35 selected variants and the list of 339 SNPs derived from our comprehensive analysis of the Colombian *Ductal Luminal* breast cancer cohort (339 SNPs included in **Supplementary Table S2**). In this matching, we found 5 common SNPs present in both sets that are: rs766301333 (in gene EPHA1, site chr7_143091418 change G to A); rs762605878 (in gene PLEKHG1, site chr6_151125863 change G to A); rs758321674 (in gene STAB2, site chr12_104100711 change G to A); rs121912656 (in gene TP53, site chr17_7577547 change C to A); and rs1057519997 (also in gene TP53, site chr17_7579355 change A to T). Along with information on SNPs, in **Supplementary Table S6** we also included information on the differential expression analysis performed for all these genes with the RNA-seq data from the 476 TCGA samples. A group of 10 genes out of 29 showed significant changes in differential expression, considering the Lima-Voom algorithm, and 25 genes out of 29, considering the DESeq2 algorithm. Many of these gene alterations observed in our analysis have been previously reported. For example, PLEKHG1 is a gene located in a breast cancer risk locus on chromosome 6, and it has been found downregulated in breast cancer samples compared to adjacent normal tissue samples [66]. We found this gene mutated and repressed. Another relevant result in our analysis was the detection of the tumor suppressor TP53 presenting 3 mutations, conserved in both the Colombian and TCGA data sets. This gene as a whole did not have a significant change in the level of expression, but when we measured eQTL, which detects changes in expression between the two alleles of a mutation site, we observed that two SNPs in TP53 (rs587781288 and rs1057519997) presented a change in expression associated with the mutation: site chr17_7578508 change C to T, p-value = 0.0634; and site chr17_7579355 change A to T, p-value = 0.0926 (**Supplementary Table S6**). The changes are not very significant but indicate a trend. In both cases, the mutation corresponded to upregulation of the gene, indicating that the mutation may have a positive effect by enhancing the tumor suppressor activity of TP53 in *Ductal Luminal* breast carcinomas. We calculated the eQTLs for all the 35 mutations included in this study, and found only 2 other cases in which the mutation is associated with a change in expression: AKT1 gene, rs121434592 mutation (site chr14_105246551 change C to T) p-value = 0.0068; and PIK3CA gene, rs121913273 mutation (site chr3_178936082 change G to A) p-value = 0.0435. These two genes, AKT1 and PIK3CA, are well-known cancer genes and we report their double alteration in sequence and in expression detected in both *Ductal Luminal* breast cancer cohorts.

4. Conclusions

In the present study we found a set of gene-centric alterations identified as pathogenic mutations in exomes from invasive breast carcinomas of *Ductal Luminal* subtype, in a cohort of patients of Southwest Colombia. The pathogenic mutations were detected as somatic tumor variants corresponding to non-synonymous single nucleotide polymorphisms (nsSNPs). The mutations were correlated with exome mutations and genome-wide expression alterations detected in a larger set of cancer samples of the same breast cancer subtype from TCGA (that included DNA-seq and RNA-seq data). The results provide a refined list of genes and mutations associated with the etiology of the *Ductal Luminal* breast cancer. This list includes 19 single mutations identified as tumor driver mutations in 17 genes. Some of the genes (such as ATM, ERBB3, ESR1 or TP53) are very well-known cancer genes altered in breast cancer and therefore expected, while other genes (such as CBLB and PRPF8) presented driver mutations that had not been reported before. Moreover, in the case of CBLB gene, 13 mutations were identified in TCGA *Ductal Luminal* samples associated with overexpression of their host gene and repression of tumor suppressive activity of TGF-Beta pathway. Our study also reports a combined analysis of WES samples from the Colombian and TCGA patients, providing a set of 29 common genes that were found mutated in both *Ductal Luminal* breast carcinoma cohorts. These genes include 35 single nucleotide mutations. Using TCGA data, we also calculated the eQTLs for all these 35 mutations, finding only 4 mutations that showed a significant change in expression

associated with the modified allele, corresponding to mutations in 3 cancer genes: AKT1, PIK3CA and TP53. The functional relevance of each of these mutations in these genes, and the molecular effect on specific tumors and individual patients needed further investigation and is beyond the scope of this work. In any case, we present a neat collection of driver genetic mutations and expression alterations associated with a specific subtype of breast cancer and linked to a Colombian cohort of patients.

Supplementary Materials: The following files are available online at www.mdpi.com associated to this article: **Supplementary Figure S1** - Multidimensional scaling (MDS) analysis of 859 samples of breast ductal cells (770 from invasive ductal breast carcinomas and 89 from normal healthy controls) using RNA-seq to obtain global expression (measuring 60,423 genes). **Supplementary Table S2** - List and descriptive parameters of the 339 SNP variants (corresponding to 304 protein coding genes) found in the tumor samples of the *Ductal Luminal* breast cancer patients studied in this work. **Supplementary Table S3** - List and descriptive parameters of the 840 genes that presented significant differential expression in the set of 510 tumor samples from TCGA that corresponded to *Ductal Luminal* breast cancer subtype. **Supplementary Table S4** - List and descriptive parameters of the 304 protein coding genes that had mutation variants in the tumor samples of the *Ductal Luminal* breast cancer patients studied in this work. The genes also include the differential expression parameters and data corresponding to the comparison of 510 *Ductal Luminal* versus 89 *Control* samples from TCGA. **Supplementary Table S5** – List and descriptive parameters of the 13 somatic mutation sites that were found in gene CBLB in the 476 exomes of IDC-LM-BRCA TCGA samples. **Supplementary Table S6** – List and descriptive parameters of the 35 mutations present in the both IDC-LM-BRCA populations: TCGA patients (476) and Colombia patients (33). Four of these 35 mutations provided fairly significant eQTLs (p.value < 0.1) and are marked in the table in the column labeled "*significant_eQTL* ".

Abbreviations: BRCA: Breast carcinoma; CGI: Cancer Genome Interpreter; CPM: Counts per million; eQTL: expression Quantitative Trait Loci; ER: Estrogen receptor; IDC: Invasive Ductal Carcinoma; ILC: Invasive Lobular Carcinoma; LM: Luminal-like breast cancer tumors; MDS: Multi-dimensional scaling; PR: Progesterone receptor; TCGA: The Cancer Genome Atlas; WES: Whole Exome Sequencing.

Author Contributions: Conceptualization, C.C-U., G.B., P.E.V-V., P.A.M. and J.D.L.R.; methodology, C.C-U., F.B-G., F.T-T., G.B., J.V-M., P.A.M. and J.D.L.R.; samples and clinical data collection, C.C-U., M.S., M.G-B., P.E.V-V., J.C.B. and G.B.; software development, application and validation, C.C-U., F.B-G., F.T-T., and J.D.L.R.; formal analysis, investigation, resources and data curation, C.C-U., F.B-G., G.B., and J.D.L.R.; writing - original draft preparation, C.C-U., F.B-G., and J.D.L.R.; writing - review and editing, C.C-U., F.B-G., M.S., M.G-B., F.T-T., P.E.V-V., J.C.B., G.B., J.V-M., P.A.M. and J.D.L.R.; supervision and project administration, G.B., J.V-M., P.A.M. and J.D.L.R.; funding acquisition, P.A.M. and J.D.L.R.

Funding: This research was funded by *Fondo de Investigación Sanitaria - Instituto de Salud Carlos III* (FIS - ISCIII, Spanish Ministry of Health, project reference PI18/00591) where J.D.L.R. was the PI. The work within this project was also co-funded by the FEDER program of the European Union. The project on breast cancer from Colombia patients was funded by *Sistema de Regalías del Valle del Cauca*, Colombia (reference BPIN 2013000100297).

Acknowledgments: : The project on breast cancer from Colombia patients was funded by *Sistema de Regalías del Valle del Cauca*, Colombia (reference BPIN 2013000100297). J.D.L.R. lab also acknowledges the support given to his group by the European Project H2020 ArrestAD (Project ID: 737390, H2020-FETOPEN-1-2016-2017).

Conflicts of Interest: All the authors declare no conflict of interest. The funders had no role in the design of the study; in the collection, analyses, or interpretation of data; in the writing of the manuscript, or in the decision to publish the results.

References

1. Zaidi, Z.; Dib, H.A. Abstract 4191: The worldwide female breast cancer incidence and survival, 2018. *Cancer Res* **2019**, *79*, 4191-4191. DOI:10.1158/1538-7445.Am2019-4191.
2. Bray, F.; Ferlay, J.; Soerjomataram, I.; Siegel, R.L.; Torre, L.A.; Jemal, A. Global cancer statistics 2018: GLOBOCAN estimates of incidence and mortality worldwide for 36 cancers in 185 countries. CA: A Cancer Journal for Clinicians 2018, 68, 394-424. DOI:10.3322/caac.21492.

3. Cortés, C.; Rivera, A.L.; Trochez, D.; Solarte, M.; Gómez, D.; Cifuentes, L.; Barreto, G. Mutational analysis of BRCA1 and BRCA2 genes in women with familial breast cancer from different regions of Colombia. Hered Cancer Clin Pract 2019, 17, 20-20. DOI:10.1186/s13053-019-0120-x.
4. Pardo, C.; de Vries, E. Breast and cervical cancer survival at Instituto Nacional de Cancerología, Colombia. Colomb Med (Cali) 2018, 49, 102-108. DOI:10.25100/cm.v49i1.2840.
5. Yersal, O.; Barutca, S. Biological subtypes of breast cancer: Prognostic and therapeutic implications. World J Clin Oncol 2014, 5, 412-424. DOI:10.5306/wjco.v5.i3.412.
6. Sharma, G.N.; Dave, R.; Sanadya, J.; Sharma, P.; Sharma, K.K. Various types and management of breast cancer: An overview. J Adv Pharm Technol Res 2010, 1, 109-126.
7. Dai, X.; Li, T.; Bai, Z.; Yang, Y.; Liu, X.; Zhan, J.; Shi, B. Breast cancer intrinsic subtype classification, clinical use and future trends. Am J Cancer Res 2015, 5, 2929-2943.
8. Urbach, D.; Lupien, M.; Karagas, M.R.; Moore, J.H. Cancer heterogeneity: origins and implications for genetic association studies. Trends Genet 2012, 28, 538-543. DOI:10.1016/j.tig.2012.07.001.
9. Chakraborty, S.; Hosen, M.I.; Ahmed, M.; Shekhar, H.U. Onco-Multi-OMICS Approach: A New Frontier in Cancer Research. Biomed Res Int 2018, 2018, 9836256-9836256. DOI:10.1155/2018/9836256.
10. Wilkerson, M.D.; Cabanski, C.R.; Sun, W.; Hoadley, K.A.; Walter, V.; Mose, L.E.; Troester, M.A.; Hammerman, P.S.; Parker, J.S.; Perou, C.M., et al. Integrated RNA and DNA sequencing improves mutation detection in low purity tumors. Nucleic Acids Res 2014, 42, e107-e107. DOI:10.1093/nar/gku489.
11. Kumar, P.; Henikoff, S.; Ng, P.C. Predicting the effects of coding non-synonymous variants on protein function using the SIFT algorithm. Nature Protocols 2009, 4, 1073-1081. DOI:10.1038/nprot.2009.86.
12. Adzhubei, I.; Jordan, D.M.; Sunyaev, S.R. Predicting functional effect of human missense mutations using PolyPhen-2. Curr Protoc Hum Genet 2013, Chapter 7, Unit7.20-Unit27.20. DOI:10.1002/0471142905.hg0720s76.
13. Schwarz, J.M.; Cooper, D.N.; Schuelke, M.; Seelow, D. MutationTaster2: mutation prediction for the deep-sequencing age. Nature Methods 2014, 11, 361-362. DOI:10.1038/nmeth.2890.
14. Shihab, H.A.; Gough, J.; Cooper, D.N.; Day, I.N.M.; Gaunt, T.R. Predicting the functional consequences of cancer-associated amino acid substitutions. Bioinformatics 2013, 29, 1504-1510. DOI:10.1093/bioinformatics/btt182.
15. Kircher, M.; Witten, D.M.; Jain, P.; O'Roak, B.J.; Cooper, G.M.; Shendure, J. A general framework for estimating the relative pathogenicity of human genetic variants. Nat Genet 2014, 46, 310-315. DOI:10.1038/ng.2892.
16. Davydov, E.V.; Goode, D.L.; Sirota, M.; Cooper, G.M.; Sidow, A.; Batzoglou, S. Identifying a high fraction of the human genome to be under selective constraint using GERP++. PLoS Comput Biol 2010, 6, e1001025-e1001025. DOI:10.1371/journal.pcbi.1001025.
17. Norris, E.T.; Wang, L.; Conley, A.B.; Rishishwar, L.; Mariño-Ramírez, L.; Valderrama-Aguirre, A.; Jordan I.K. Genetic ancestry, admixture and health determinants in Latin America. *BMC Genomics* **2018**, *19*, 861. DOI:10.1186/s12864-018-5195-7.
18. Colaprico, A.; Silva, T.C.; Olsen, C.; Garofano, L.; Cava, C.; Garolini, D.; Sabedot, T.S.; Malta, T.M.; Pagnotta, S.M.; Castiglioni, I., et al. TCGAbiolinks: an R/Bioconductor package for integrative analysis of TCGA data. Nucleic Acids Res 2016, 44, e71-e71. DOI:10.1093/nar/gkv1507.
19. Silva, T.C.; Colaprico, A.; Olsen, C.; D'Angelo, F.; Bontempi, G.; Ceccarelli, M.; Noushmehr, H. TCGA Workflow: Analyze cancer genomics and epigenomics data using Bioconductor packages. F1000Res 2016, 5, 1542-1542. DOI:10.12688/f1000research.8923.2.
20. Chen, Y.; Lun, A.T.L.; Smyth, G.K. From reads to genes to pathways: differential expression analysis of RNA-Seq experiments using Rsubread and the edgeR quasi-likelihood pipeline. F1000Res 2016, 5, 1438-1438. DOI:10.12688/f1000research.8987.2.
21. Robinson, M.D.; Oshlack, A. A scaling normalization method for differential expression analysis of RNA-seq data. Genome Biol 2010, 11, R25-R25. DOI:10.1186/gb-2010-11-3-r25.
22. Law, C.W.; Chen, Y.; Shi, W.; Smyth, G.K. voom: precision weights unlock linear model analysis tools for RNA-seq read counts. Genome Biol 2014, 15, R29. DOI:10.1186/gb-2014-15-2-r29.
23. Love, M.I.; Huber, W.; Anders, S. Moderated estimation of fold change and dispersion for RNA-seq data with DESeq2. Genome Biol 2014, 15, 550-550. DOI:10.1186/s13059-014-0550-8.
24. Benjamini, Y.; Hochberg, Y. Controlling the False Discovery Rate: A Practical and Powerful Approach to Multiple Testing. Journal of the Royal Statistical Society. Series B (Methodological) 1995, 57, 289-300.

25. Tamborero, D.; Rubio-Perez, C.; Deu-Pons, J.; Schroeder, M.P.; Vivancos, A.; Rovira, A.; Tusquets, I.; Albanell, J.; Rodon, J.; Tabernero, J., et al. Cancer Genome Interpreter annotates the biological and clinical relevance of tumor alterations. Genome Med 2018, 10, 25-25. DOI:10.1186/s13073-018-0531-8.
26. Schroeder, M.P.; Rubio-Perez, C.; Tamborero, D.; Gonzalez-Perez, A.; Lopez-Bigas, N. OncodriveROLE classifies cancer driver genes in loss of function and activating mode of action. Bioinformatics 2014, 30, i549-i555. DOI:10.1093/bioinformatics/btu467.
27. Mularoni, L.; Sabarinathan, R.; Deu-Pons, J.; Gonzalez-Perez, A.; López-Bigas, N. OncodriveFML: a general framework to identify coding and non-coding regions with cancer driver mutations. Genome Biol 2016, 17, 128. DOI:10.1186/s13059-016-0994-0.
28. Lever, J.; Zhao, E.Y.; Grewal, J.; Jones, M.R.; Jones, S.J.M. CancerMine: a literature-mined resource for drivers, oncogenes and tumor suppressors in cancer. Nature Methods 2019, 16, 505-507. DOI:10.1038/s41592-019-0422-y.
29. The UniProt, C. UniProt: a worldwide hub of protein knowledge. Nucleic Acids Res 2018, 47, D506-D515. DOI:10.1093/nar/gky1049.
30. Tate, J.G.; Bamford, S.; Jubb, H.C.; Sondka, Z.; Beare, D.M.; Bindal, N.; Boutselakis, H.; Cole, C.G.; Creatore, C.; Dawson, E., et al. COSMIC: the Catalogue Of Somatic Mutations In Cancer. Nucleic Acids Res 2019, 47. D941-D947, DOI:10.1093/nar/gky1015.
31. Griffith, M.; Spies, N.C.; Krysiak, K.; McMichael, J.F.; Coffman, A.C.; Danos, A.M.; Ainscough, B.J.; Ramirez, C.A.; Rieke, D.T.; Kujan, L., et al. CIViC is a community knowledgebase for expert crowdsourcing the clinical interpretation of variants in cancer. Nat Genet 2017, 49, 170-174. DOI:10.1038/ng.3774.
32. Ainscough, B.J.; Griffith, M.; Coffman, A.C.; Wagner, A.H.; Kunisaki, J.; Choudhary, M.N.; McMichael, J.F.; Fulton, R.S.; Wilson, R.K.; Griffith, O.L., et al. DoCM: a database of curated mutations in cancer. Nat Methods 2016, 13, 806-807. DOI:10.1038/nmeth.4000.
33. Landrum, M.; Lee, J.; Benson, M.; Brown, G.; Chao, C.; Chitipiralla, S.; Gu, B.; Hart, J.; Hoffman, D.; Hoover, J., et al. ClinVar: Public archive of interpretations of clinically relevant variants. Nucleic Acids Res 2015, 44. DOI:10.1093/nar/gkv1222.
34. Chakravarty, D.; Gao, J.; Phillips, S.; Kundra, R.; Zhang, H.; Wang, J.; Rudolph, J.E.; Yaeger, R.; Soumerai, T.; Nissan, M.H., et al. OncoKB: A Precision Oncology Knowledge Base. JCO Precis Oncol 2017, 10.1200/PO.17.00011, 1-16. DOI:10.1200/PO.17.00011.
35. Repana, D.; Nulsen, J.; Dressler, L.; Bortolomeazzi, M.; Venkata, S.K.; Tourna, A.; Yakovleva, A.; Palmieri, T.; Ciccarelli, F.D. The Network of Cancer Genes (NCG): a comprehensive catalogue of known and candidate cancer genes from cancer sequencing screens. Genome Biol 2019, 20, 1. DOI:10.1186/s13059-018-1612-0.
36. Szklarczyk, D.; Franceschini, A.; Wyder, S.; Forslund, K.; Heller, D.; Huerta-Cepas, J.; Simonovic, M.; Roth, A.; Santos, A.; Tsafou, K.P., et al. STRING v10: protein-protein interaction networks, integrated over the tree of life. Nucleic Acids Res 2015, 43, D447-D452. DOI:10.1093/nar/gku1003.
37. Alonso-López, D.; Gutiérrez, M.A.; Lopes, K.P.; Prieto, C.; Santamaría, R.; De Las Rivas, J. APID interactomes: providing proteome-based interactomes with controlled quality for multiple species and derived networks. *Nucleic Acids Res* **2016**, *44(W1)*, W529-35. DOI:10.1093/nar/gkw363.
38. Fontanillo, C.; Nogales-Cadenas, R.; Pascual-Montano, A.; De Las Rivas, J. Functional Analysis beyond Enrichment: Non-Redundant Reciprocal Linkage of Genes and Biological Terms. PloS One 2011, 6, e24289. DOI:10.1371/journal.pone.0024289.
39. Conley, A.B.; Rishishwar, L.; Norris, E.T.; Valderrama-Aguirre, A.; Mariño-Ramírez, L.; Medina-Rivas, M.A.; Jordan, I.K. A Comparative Analysis of Genetic Ancestry and Admixture in the Colombian Populations of Chocó and Medellín. G3 (Bethesda) 2017, 7, 3435-3447. DOI:10.1534/g3.117.1118.
40. Neelamraju, Y.; Gonzalez-Perez, A.; Bhat-Nakshatri, P.; Nakshatri, H.; Janga, S.C. Mutational landscape of RNA-binding proteins in human cancers. RNA Biol 2018, 15, 115-129. DOI:10.1080/15476286.2017.1391436.
41. Kang, J.M.; Park, S.; Kim, S.J.; Hong, H.Y.; Jeong, J.; Kim, H.S; Kim, S.J. CBL enhances breast tumor formation by inhibiting tumor suppressive activity of TGF-β signaling. Oncogene 2012, 50, 5123-5131. DOI: 10.1038/onc.2012.18
42. Popp, M.W.; Maquat, L.E. Nonsense-mediated mRNA Decay and Cancer. Curr Opin Genet Dev 2018, 48, 44-50. DOI:10.1016/j.gde.2017.10.007.
43. Perrin-Vidoz, L.; Sinilnikova, O.M.; Stoppa-Lyonnet, D.; Lenoir, G.M.; Mazoyer, S. The nonsense-mediated mRNA decay pathway triggers degradation of most BRCA1 mRNAs bearing premature termination codons. Hum Mol Genet 2002, 11, 2805-2814. DOI:10.1093/hmg/11.23.2805.

44. Villa, E.; Ali, E.S.; Sahu, U.; Ben-Sahra, I. Cancer Cells Tune the Signaling Pathways to Empower de Novo Synthesis of Nucleotides. Cancers (Basel) 2019, 11, 688. DOI:10.3390/cancers11050688.
45. Ring, A.E.; Smith, I.E.; Ashley, S.; Fulford, L.G.; Lakhani, S.R. Oestrogen receptor status, pathological complete response and prognosis in patients receiving neoadjuvant chemotherapy for early breast cancer. Br J Cancer 2004, 91, 2012-2017. DOI:10.1038/sj.bjc.6602235.
46. Rouzier, R.; Perou, C.M.; Symmans, W.F.; Ibrahim, N.; Cristofanilli, M.; Anderson, K.; Hess, K.R.; Stec, J.; Ayers, M.; Wagner, P., et al. Breast Cancer Molecular Subtypes Respond Differently to Preoperative Chemotherapy. Clin Cancer Res 2005, 11, 5678-5685. DOI:10.1158/1078-0432.CCR-04-2421.
47. Boon, K.-L.; Norman, C.M.; Grainger, R.J.; Newman, A.J.; Beggs, J.D. Prp8p dissection reveals domain structure and protein interaction sites. RNA 2006, 12, 198-205. DOI:10.1261/rna.2281306.
48. Kurtovic-Kozaric, A.; Przychodzen, B.; Singh, J.; Konarska, M.M.; Clemente, M.J.; Otrock, Z.K.; Nakashima, M.; Hsi, E.D.; Yoshida, K.; Shiraishi, Y., et al. PRPF8 defects cause missplicing in myeloid malignancies. Leukemia 2015, 29, 126-136. DOI:10.1038/leu.2014.144.
49. Henry, N.L.; Hayes, D.F. Cancer biomarkers. Mol Oncol 2012, 6, 140-146. DOI:10.1016/j.molonc.2012.01.010.
50. Toy, W.; Weir, H.; Razavi, P.; Lawson, M.; Goeppert, A.U.; Mazzola, A.M.; Smith, A.; Wilson, J.; Morrow, C.; Wong, W.L., et al. Activating ESR1 Mutations Differentially Affect the Efficacy of ER Antagonists. Cancer Discov 2017, 7, 277-287. DOI:10.1158/2159-8290.CD-15-1523.
51. Jeselsohn, R.; Yelensky, R.; Buchwalter, G.; Frampton, G.; Meric-Bernstam, F.; Gonzalez-Angulo, A.M.; Ferrer-Lozano, J.; Perez-Fidalgo, J.A.; Cristofanilli, M.; Gómez, H., et al. Emergence of constitutively active estrogen receptor-α mutations in pretreated advanced estrogen receptor-positive breast cancer. Clin Cancer Res 2014, 20, 1757-1767. DOI:10.1158/1078-0432.CCR-13-2332.
52. Robinson, D.R.; Kalyana-Sundaram, S.; Wu, Y.-M.; Shankar, S.; Cao, X.; Ateeq, B.; Asangani, I.A.; Iyer, M.; Maher, C.A.; Grasso, C.S., et al. Functionally recurrent rearrangements of the MAST kinase and Notch gene families in breast cancer. Nat Med 2011, 17, 1646-1651. DOI:10.1038/nm.2580.
53. Lei, J.T.; Gou, X.; Seker, S.; Ellis, M.J. ESR1 alterations and metastasis in estrogen receptor positive breast cancer. J Cancer Metastasis Treat 2019, 5, 38. DOI:10.20517/2394-4722.2019.12.
54. Jeselsohn, R.; Bergholz, J.S.; Pun, M.; Cornwell, M.; Liu, W.; Nardone, A.; Xiao, T.; Li, W.; Qiu, X.; Buchwalter, G., et al. Allele-Specific Chromatin Recruitment and Therapeutic Vulnerabilities of ESR1 Activating Mutations. Cancer Cell 2018, 33, 173-186.e5. DOI:10.1016/j.ccell.2018.01.004.
55. Fujiwara, S.; Yamamoto-Ibusuki, M.; Yamamoto, S.; Yamamoto, Y.; Iwase, H. Association of ErbB1-4 expression in invasive breast cancer with clinicopathological characteristics and prognosis. Breast Cancer 2014, 21, 472-481. DOI:10.1007/s12282-012-0415-5.
56. Wang, S.; Huang, X.; Lee, C.K.; Liu, B. Elevated expression of erbB3 confers paclitaxel resistance in erbB2-overexpressing breast cancer cells via upregulation of Survivin. Oncogene 2010, 29, 4225-4236. DOI:10.1038/onc.2010.180.
57. Balko, J.M.; Miller, T.W.; Morrison, M.M.; Hutchinson, K.; Young, C.; Rinehart, C.; Sánchez, V.; Jee, D.; Polyak, K.; Prat, A., et al. The receptor tyrosine kinase ErbB3 maintains the balance between luminal and basal breast epithelium. Proc Natl Acad Sci U S A 2012, 109, 221-226. DOI:10.1073/pnas.1115802109.
58. Sun, Y.-S.; Zhao, Z.; Yang, Z.-N.; Xu, F.; Lu, H.-J.; Zhu, Z.-Y.; Shi, W.; Jiang, J.; Yao, P.-P.; Zhu, H.-P. Risk Factors and Preventions of Breast Cancer. Int J Biol Sci 2017, 13, 1387-1397. DOI:10.7150/ijbs.21635.
59. Morrison, D.K. MAP kinase pathways. Cold Spring Harb Perspect Biol 2012, 4, a011254. DOI:10.1101/cshperspect.a011254.
60. Chambard, J.-C.; Lefloch, R.; Pouysségur, J.; Lenormand, P. ERK implication in cell cycle regulation. *Biochim Biophys Acta - Mol Cell Res* **2007**, *1773*, 1299-1310. DOI: 10.1016/j.bbamcr.2006.11.010.
61. Luo, J.; Manning, B.D.; Cantley, L.C. Targeting the PI3K-Akt pathway in human cancer: Rationale and promise. *Cancer Cell* **2003**, *4*, 257-262. DOI: 10.1016/S1535-6108(03)00248-4.
62. Barnes, D.M.; Millis, R.R.; Gillett, C.E.; Ryder, K.; Skilton, D.; Fentiman, I.S.; Rubens, R.D. The interaction of oestrogen receptor status and pathological features with adjuvant treatment in relation to survival in patients with operable breast cancer: a retrospective study of 2660 patients. *Endocr Relat Cancer* **2004**, *11*, 85-96. DOI: 10.1677/erc.0.0110085.
63. Nadal, A.; Ropero, A.B.; Laribi, O.; Maillet, M.; Fuentes, E.; Soria, B. Nongenomic actions of estrogens and xenoestrogens by binding at a plasma membrane receptor unrelated to estrogen receptor alpha and estrogen receptor beta. *Proc Natl Acad Sci U S A* **2000**, *97*, 11603-11608. DOI: 10.1073/pnas.97.21.11603.

64. Rhana, P.; Trivelato Junior, R.R.; Beirão, P.S.L.; Cruz, J.S.; Rodrigues, A.L.P. Is there a role for voltage-gated Na+ channels in the aggressiveness of breast cancer?. *Braz J Med Biol Res* **2017**, *50*, e6011. DOI: 10.1590/1414-431X20176011.
65. Rao, V.R.; Perez-Neut, M.; Kaja, S.; Gentile, S. Voltage-gated ion channels in cancer cell proliferation. *Cancers* **2015**, *7*, 849-875. DOI: 10.3390/cancers7020813.
66. Sun, Y.; Ye, C.; Guo, X.; Wen, W.; Long, J.; Gao, Y.T.; Shu, X.O.; Zheng, W.; Cai, Q. Evaluation of potential regulatory function of breast cancer risk locus at 6q25.1. *Carcinogenesis* **2016**, *37*, 163-168. DOI: 10.1093/carcin/bgv170..

© 2019 by the authors. Submitted for possible open access publication under the terms and conditions of the Creative Commons Attribution (CC BY) license (http://creativecommons.org/licenses/by/4.0/).
