# Supplementary figures and images for: Exomes of Ductal Luminal Breast Cancer Patients from Southwest Colombia: Gene Mutational Profile and Related Expression Alterations
Source: Biomolecules. 2020 Apr 30;10(5):698. doi: 10.3390/biom10050698 (PMC7277822; doi:10.3390/biom10050698)

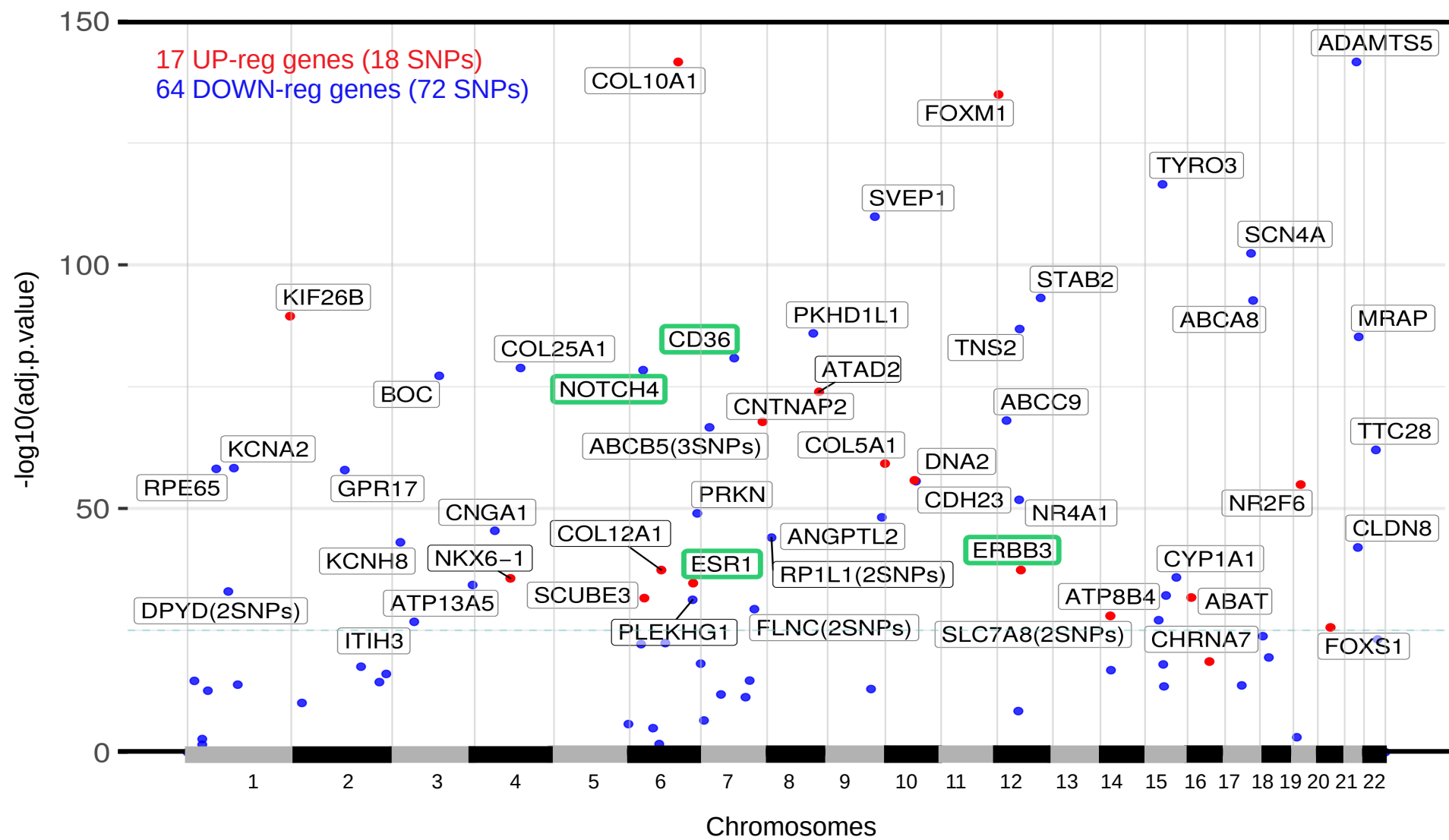

Supplement: Supplementary file 1 [file biomolecules-10-00698-s001.zip › Article-to-BIOMOLECULES_R1_v4Apr2020/CortesUrreaetal_R1_Figure_3.pdf]
