# Supplementary material for: Exomes of Ductal Luminal Breast Cancer Patients from Southwest Colombia: Gene Mutational Profile and Related Expression Alterations
Source: Biomolecules. 2020 Apr 30;10(5):698. doi: 10.3390/biom10050698 (PMC7277822; doi:10.3390/biom10050698)
Supplement: Supplementary file 1 [file CortesUrreaetal_R1_Biomolecules707977_ResponseLetter.pdf › Article-to-BIOMOLECULES_R1_v4Apr2020/CortesUrreaetal_R1_Biomolecules707977_ResponseLetter.pdf]

Response Letter

Journal: **Biomolecules**
Manuscript ID: **biomolecules-707977**

Dear Editor,

We sent the revised version of our manuscript entitled "**Exomes of *Ductal Luminal* breast cancer patients from Southwest Colombia: gene mutational profile and related expression alterations**" by *Carolina Cortes-Urrea, Fernando Bueno-Gutierrez, Melissa Solarte, Miguel Guevara-Burbano, Fabian Tobar-Tosse, Patricia E. Vélez-Varela, Juan Carlos Bonilla, Guillermo Barreto, Pedro A. Moreno and Javier De Las Rivas*. As indicated by the reviewers and the editor we have done a major revision of our article addressing point by point all the comments and questions that the reviewers formulated. We enclose below our responses, indicating also all the parts where the manuscript has been modified.

As we indicated in our first submission, please consider our article for publication in the *Special Issue* "**Bioinformatics and Precision Computational Biology: Selected Papers from the X International Conference on Bioinformatics #SolBio+10**", that belongs to the section of *Bioinformatics and Systems Biology* of the journal ***Biomolecules***.

Best regards,
Dr. Javier De Las Rivas (jrivas@usal.es)
Bioinformatics and Functional Genomics
Cancer Research Center (CiC-IBMCC, CSIC/USAL/IBSAL)
Consejo Superior de Investigaciones Científicas (CSIC) & University of Salamanca (USAL)
Salamanca (Spain)

Reviewer 1

In the manuscript "Exomes of Ductal Luminal breast cancer patients from Southwest Colombia: gene mutational profile and related expression alterations", the authors tried to discover the driver genes of malignant transformations in breast cancer patients. They first collected the mutational genomic profile by whole exome sequencing (WES) in a set of cancer samples from southwest Colombian women. Then they combined this with the deferential gene expression (DEG) data from the same breast cancer subtype. They finally identified the genes with significant changes in both the mutational profile and the expression profile.

Overall, the study presented is technically sound and their results are valuable for the patients. …

Response 1.1. – Thank you for the general comment and the understanding of the work.

However, using RNA-seq data from TCGA to correspond their WES data is not sufficient since the similarity of their samples to the samples in the TCGA database is not clear.

Response 1.2. – Thank you for this point. It is true, as the reviewer indicates, that in our manuscript "the similarity of their samples to the samples in the TCGA database is not clear". To address this, we have included in our manuscript a more detailed explanation of the similarities between the patients from Colombia and the subset of TCGA samples used in our analyses. So, on **pages 3-4**, **lines 140-171**, the similarities are explained:

2.5. Selection of samples from TCGA for comparative analysis with Colombian samples

We assessed the clinical characteristics of our cohort of Colombian patients to select a similar cohort of patients from TCGA and study them together. In this selection we pursue a series of clinical and phenotypic similarities between these two cohorts of patients to allow that the in-house Colombian WES data could be compared with WES and RNA-seq data from TCGA. As indicated above, in order to do this comparative analysis of genetic mutations (somatic variants) and expression data, it was very important to previously select specific cancer subtypes. Since most of the Colombian patients were *Ductal* and *Luminal* (33/52, 63.5%), our study focused on the analysis of this specific cancer subtype: *Invasive Ductal - Luminal - Breast Cancer* (IDC-LM-BRCA). Therefore, considering the characteristics of the Colombian samples we did a selection of a similar set of samples from TCGA. These similarities were the following:

(i) All patients from Colombia and from TCGA selection were women of similar age: presenting an average of 61.6 years old at diagnosis (standard deviation ± 12.6) for the Colombian cohort; and average of 57.3 years (SD ± 13.2) for the selected TCGA patients.

(ii) Both cohorts of patients were mostly White. A recent genetic study by Norris et al., 2017 [17] stated that the population from Antioquia, a close Colombian state culturally very similar to the patient's region (Valle del Cauca), shows averages of: 64% European ancestry, 29% Native American ancestry and 7% African ancestry. The majority of the selected TCGA patients were also White of European Ancestry (496/770, 64%). Therefore, to a large extent the Colombian and the TCGA patients have a similar genetic background. The remaining TCGA patients were: Black or African American (148/770, 19.2%), Asian (47/770, 6%), American Indian or Alaska Native (1/770, 0.01%), and not reported race (78/770, 10%).

(iii) With respect to the cellular subtypes, all the breast cancer patients selected from TCGA were *Invasive Ductal Carcinoma*. In this way, we matched with the main cellular subtype of the WES samples from Colombia: 42/52 (81%) *Invasive Ductal Carcinoma* (IDC).

(iv) With respect to the breast cancer intrinsic subtypes, the whole set of 770 tumor samples from TCGA were: *Luminal A* (339), *Luminal B* (171), *Basal* (165), *Her2* (73) and *Normal* (22). For the comparison with the Colombian cohort we only used the *Luminal* samples (339 + 171 = 510), because the majority of the Colombian samples (within the *Ductal*) were of *Luminal* subtype.

(v) Tumor stage: in both groups of patients, the majority of the samples corresponded to stage I and II tumors: 81% of the Colombian patients, and 76% of the patients selected from TCGA. Furthermore, none of the in-house patients from Colombia or TCGA patients had metastases.

Response 1.3. – Also to respond to this concern of the reviewer saying that "using RNA-seq data from TCGA … is not sufficient ", we have included new WES data from TCGA to do a better comparative analysis with the WES data from Colombia. This is explained in a new section of the manuscript on **page 6**, **lines 244-261**:

2.10. Combined analysis of WES data from the Colombian and TCGA cohorts

To complement the approach illustrated in **Figure 1**, we used the WES data available for breast cancer patients from TCGA that were included in the expression analysis. Using this WES data from TCGA we searched and identified mutations that were present also in the Colombian populations. The WES data (hg38/NCBI GRCh38) including somatic mutations of the primary tumors of 713 patients with *Invasive Ductal Carcinoma* from TCGA project were downloaded from GDC data portal. The data contained 79,508 different mutation sites. As indicated above, the dataset was filtered to include only the 476 samples from the patients of *Ductal Luminal breast cancer* subtype (IDC-LM-BRCA) (476/713, 67%). Within these samples the number of mutations sites found was 43,213. Moreover, all these 476 patients were within the set of 510 *Ductal Luminal breast cancer* patients used in the differential expression analysis (**Figure 1**). Once defined the specific tumor subtype studied, we combined the Colombian and the TCGA datasets (i.e., 33 and 476 WES samples, respectively) to search for their common mutation sites. The WES data from Colombia were prepared as explained in sections 2.2 and 2.3. However, to achieve a better comparison of both WES data, the pathogenicity filters were not taken into account, since such pathogenic information was not available in the same way for the samples from TCGA. Therefore, we took the Colombian WES data prior to the application of the filters (which contained 45,454 mutation sites) and combined it with the 43,213 mutation sites found in the TCGA WES data, to find the intersection of both sets.

This new combined analysis of WES samples from Colombia and TCGA provided a set of 29 common genes that were found mutated in both cohorts of *Ductal Luminal* breast cancer

patients. These genes include 35 single nucleotide mutations, with the following three genes exhibiting multiple mutations: PIK3CA with 4 mutations, TP53 with 3 mutations, and MUC4 with 2 mutations. Using the data from TCGA, we also calculated eQTLs (expression Quantitative Trait Loci) for all these 35 mutations, finding only 4 significative that are reported. All this information is included in an additional supplementary table in the updated manuscript (**Supplementary Table S6**). We also present these results in a new paragraph of the manuscript on **page 13-14**, **lines 572-613**:

3.8. Common mutated genes in Ductal Luminal breast cancer patients from Colombia and TCGA

As described in section 2.10. (*Combined analysis of WES data from the Colombian and TCGA cohorts*) we performed the intersection of 43,213 mutation sites found in the 476 TCGA *Ductal Luminal* patients and the 45,454 mutation sites in the 33 Colombia *Ductal Luminal* patients. This combined analysis of WES samples from Colombia and TCGA provided a set of 29 common genes that were found mutated in both cohorts of *Ductal Luminal* breast carcinoma patients. These genes include 35 single nucleotide mutations, with the following 3 genes exhibiting multiple mutations: PIK3CA with 4 mutations, TP53 with 3, and MUC4 with 2. PIK3CA mutations represent one of the most common genetic aberrations in breast cancer. They have been reported to be present in over one-third of cases, with enrichment in the *luminal* subtypes. The tumor suppressor gene TP53 is the most frequently mutated gene in somatic cells of human cancers. All the information on these mutations is included in an additional supplementary table called: **Supplementary Table S6**. Along with the genes that overlap between the Colombian and TCGA datasets, we also looked for the specific mutations that matched between this list of 35 selected variants and the list of 339 SNPs derived from our comprehensive analysis of the Colombian *Ductal Luminal* breast cancer cohort (339 SNPs included in **Supplementary Table S2**). In this matching, we found 5 common SNPs present in both sets that are: rs766301333 (in gene EPHA1, site chr7_143091418 change G to A); rs762605878 (in gene PLEKHG1, site chr6_151125863 change G to A); rs758321674 (in gene STAB2, site chr12_104100711 change G to A); rs121912656 (in gene TP53, site chr17_7577547 change C to A); and rs1057519997 (also in gene TP53, site chr17_7579355 change A to T). Along with information on SNPs, in **Supplementary Table S6** we also included information on the differential expression analysis performed for all these genes with the RNA-seq data from the 476 TCGA samples. A group of 10 genes out of 29 showed significant changes in differential expression, considering the Lima-Voom algorithm, and 25 genes out of 29, considering the DESeq2 algorithm. Many of these gene alterations observed in our analysis have been previously reported. For example, PLEKHG1 is a gene located in a breast cancer risk locus on chromosome 6, and it has been found downregulated in breast cancer samples compared to adjacent normal tissue samples [66]. We found this gene mutated and repressed. Another relevant result in our analysis was the detection of the tumor suppressor TP53 presenting 3 mutations, conserved in both the Colombian and TCGA data sets. This gene as a whole did not have a significant change in the level of expression, but when we measured eQTL, which detects changes in expression between the two alleles of a mutation site, we observed that two SNPs in TP53 (rs587781288 and rs1057519997) presented a change in expression associated with the mutation: site chr17_7578508 change C to T, p-value = 0.0634; and site chr17_7579355 change A to T, p-value = 0.0926 (**Supplementary Table S6**). The changes are not very significant but indicate a trend. In both cases, the mutation corresponded to upregulation of the gene, indicating that the mutation may have a positive effect by enhancing the tumor suppressor activity of TP53 in *Ductal Luminal* breast carcinomas. We calculated the eQTLs for all the 35 mutations included in this study, and found only 2 other cases in which the mutation is associated with a change in expression: AKT1 gene, rs121434592 mutation (site chr14_105246551 change C to T) p-value = 0.0068; and PIK3CA gene, rs121913273 mutation (site chr3_178936082 change G to A) p-value = 0.0435. These two genes, AKT1 and PIK3CA, are well-known cancer genes and we report their double alteration in sequence and in expression detected in both *Ductal Luminal* breast cancer cohorts.

Moreover, some additional findings were included in the manuscript regarding mutations found in the CBLB gene. This is explained in the new ***section 3.7.*** entitled "*Mutations found in CBLB, a gene that inhibits the TGF-Beta pathway*", that is on **page 13, lines 550-571** and also in the new **Supplementary Table S5.**

To make their conclusions reliable, it is recommended that the authors introduce more RNAseq data from different databases.

Response 1.4. – We agree with the reviewer that more RNA-seq data from other studies from other databases could strengthen our conclusions. However, we consider that the analysis of 476 TCGA samples from the same breast cancer subtype (including both WES data, obtained by DNA-seq, and global expression data, obtained by RNA-seq) is good enough to support our conclusions. In addition, we indicate more clearly in the manuscript the similarities between the TCGA and the Colombian patients (explained in detail on **pages 3-4, lines 140-171**); and this also supports to our observations and conclusions.

To conclude this point, as noted above, the analysis of whole exomes of similar breast cancer patients from Colombia and from TCGA (included in the **new manuscript**) reinforces our argument that WES data can be combined to identify more reliable common genetic alterations. Furthermore, the integration of DNA-seq with RNA-seq data from TCGA samples, also presented in the **new manuscript**, provides support for the identification of genes that have a significant alteration of the expression that is concomitant with the genetic alteration observed in the WES data.

Reviewer 2

Cortes-Urrea et al. report the overlap of genes with somatic SNP and differential expression levels in ductal luminal breast cancers. To obtain this list of genes, Cortes-Urrea et al. has subjected 52 breast cancer samples (where the majority were ductal luminal type) to whole-exome-sequencing (WES) and around 800 transcriptomes (>500 ductal luminal subtype) from the online TCGA repository. The analytic methods are well-known, and the paper is well-written.

Response 2.1.– Thanks for understanding the work and for the comment on the quality of the writing.

However, the objective of the study together with the selected material and methods do not support the conclusion which again is rather weak: "The results provide a refined list 499 of 81 genes most probably associated with the etiology of the disease in this part of Latin America. 500 Some of the genes (such as ESR1) are very well known and therefore expected……". Taken together there are some major concerns which could weaken the scientific merit.

Points of concern:

The objective and conclusions are very hard to understand in a scientific context.

Response 2.2.– We agree that the objective and conclusions of the work should be more clearly defined. We have re-written a large part of the *Introduction* and the *Conclusions* sections in order to provide a better description of the objectives of the study as well as the conclusions derived from the work done. We include below two paragraphs to show this.

On **page 2**, **lines 79-87**:

> As a whole, the objective of this study was to find a set of gene-centered alterations identified as pathogenic mutations in exomes from invasive breast carcinomas of *Ductal Luminal* subtype, in a cohort of patients of Southwest Colombia; and compare that set with a similar, but larger, cohort of TCGA patients. Pathogenic mutations were detected as somatic tumor variants corresponding to non-synonymous single nucleotide polymorphisms (nsSNPs). The results provide valuable information on the characteristics of this type of breast cancer and allow us to identify novel associations between genetic variants and related genes in *Ductal Luminal* breast carcinomas. The work revealed well-known *cancer driver* genes, along with some new *cancer driver* mutations associated with the patient subpopulation we studied.

On **pages 14-15**, **lines 614-637**:

4. Conclusions

In the present study we found a set of gene-centric alterations identified as pathogenic mutations in exomes from invasive breast carcinomas of *Ductal Luminal* subtype, in a cohort of patients of Southwest Colombia. The pathogenic mutations were detected as somatic tumor variants corresponding to non-synonymous single nucleotide polymorphisms (nsSNPs). The mutations were correlated with exome mutations and genome-wide expression alterations detected in a larger set of cancer samples of the same breast cancer subtype from TCGA (that included DNA-seq and RNA-seq data). The results provide a refined list of genes and mutations associated with the etiology of the *Ductal Luminal* breast cancer. This list includes 19 single mutations identified as tumor driver mutations in 17 genes. Some of the genes (such as ATM, ERBB3, ESR1 or TP53) are very well-known cancer genes altered in breast cancer and therefore expected, while other genes (such as CBLB and PRPF8) presented driver mutations that had not been reported before. Moreover, in the case of CBLB gene, 13 mutations were identified in TCGA *Ductal Luminal* samples associated with overexpression of their host gene and repression of tumor suppressive activity of TGF-Beta pathway. Our study also reports a combined analysis of WES samples from the Colombian and TCGA patients, providing a set of 29 common genes that were found mutated in both *Ductal Luminal* breast carcinoma cohorts. These genes include 35 single nucleotide mutations. Using TCGA data, we also calculated the eQTLs for all these 35 mutations, finding only 4 mutations that showed a significant change in expression associated with the modified allele, corresponding to mutations in 3 cancer genes: AKT1, PIK3CA and TP53. … … … In any case, we present a neat collection of driver genetic mutations and expression alterations associated with a specific subtype of breast cancer and linked to a Colombian cohort of patients.

Why did the authors not obtain the WES data from TCGA instead of costly in-house sample processing? Especially, since none of the clinical annotations from in-house patients are taken into considerations.

Response 2.3.– Thanks for this comment. We agree with the reviewer that the WES data from TCGA is very valuable for this research and, following this recommendation, we have included in our work the analysis of 476 WES samples from TCGA corresponding to *Ductal Luminal* breast cancer patients and compared them with the WES from the Colombian cohort. The description of these data, the analysis and the results are included in the new manuscript in several places:

On **page 6**, **lines 244-261**:

2.10. Combined analysis of WES data from the Colombian and TCGA cohorts

… … …

On **pages 13-14**, **lines 572-613**:

3.8. Common mutated genes in Ductal Luminal breast cancer patients from Colombia and TCGA

… … …

Finally, on this comment of the reviewer, it is also true that "clinical annotations from in-house patients" were not clearly presented in the manuscript. However, they were taken into consideration in the analysis and an explanation of the **characteristics of the Colombian samples and their similarities** (in the comparison with the WES samples taken from TCGA) is now included in the new manuscript on **pages 3-4**, **lines 140-171**, (this is also indicated to reviewer 1 above in **Response 1.2.**).

Since [52] is a rather limited number of samples for WES, I wonder why no manual curation and removal of false-positive SNP was carried out.

Response 2.4.– Manual curation and removal of false positive SNPs was done and it is explained in ***section 2.3.*** and ***2.4.*** of the manuscript (***2.3.*** *Exome mapping and genetic variant*

calling and ***2.4.*** *Variant prioritization based on greater harmful impact on protein*). We have revised this part of the manuscript to make a clearer description of the procedure we followed in the selection of the SNPs. As shown below, a step by step explanation is now included in the new manuscript on **page 3, lines 110-139**:

2.3. Exome mapping and genetic variant calling

… … …

In the analysis of the raw exome sequences, a coverage threshold of 100x was applied in order to have a clear mapping of each genomic locus and to identify well all the variants found. Statistical analysis and quality scores were computed for each single nucleotide variant, and variants with low scores were removed. The consensus of the three tools indicated above (GATK, SAMtools and FreeBayes) was taken into account for each variant, and if there was not complete agreement between them after manual inspection, the variant was not considered. Variant annotation (looking for *non-synonymous single nucleotide polymorphisms*, nsSNPs) was performed with ANNOVAR. Data from 1000 Genomes, dbSNP, ExAC (Exome Aggregation Consortium) and specially data from COSMIC (Catalogue of Somatic Mutations in Cancer) were used in the annotation of variants. In this way, after all these steps, we found a first raw set of 60,026 variants (SNPs) in 14,634 genes. Variants present in any of the 7 exomes of the control breast tissue samples were discarded, producing a second set of 41,404 variants. See steps 1 to 4, in **Figure 1**, which presents the workflow that we used to select variants. In a third filter step, each of the 41,404 variants were evaluated for the damaging / pathogenic effect on the corresponding gene using 6 different tools: SIFT [11], PolyPhen-2 [12], MutationTaster 2 [13], FATHMM [14], CADD [15] and GERP++ [16]. Each of them was used with the following pathogenity thresholds: SIFT <0.05; PolyPhen-2 >=0.98; MutationTaster A or D; FATHMM D; CADD >=20; and GERP++ >=2. This provided a strict filtering that resulted in the identification of 1,079 pathogenic variants present in 845 genes.

2.4. Variant prioritization based on greater harmful impact on protein

In order to focus on the best predictions about the functional effects of the nsSNPs in the corresponding gene-products and their identification as somatic mutations, we applied some more stringent filters. Once the 1,079 variants were identified, the 10% less pathogenic variants for each of 4 quantitative tools (SIFT, PolyPhen-2, CADD and GERP++) were discarded (step 4 in **Figure 1**). In an alternative fourth step (step 4' in **Figure 1**), we removed the 20% less pathogenic variants for each of the 4 quantitative pathogenity predictor methods (also starting from the 1,079 pathogenic variants). This last step provides only the consensus variants that were above the threshold for all four methods. This led to the identification of 508 highly pathogenic mutations finally selected, identified in the 52 exomes analysed and present in 432 genes. For each variant, we had the specific pathogenicity values provided by the tools, therefore, these 508 variants were ranked from more to less confident. This final set represents the most significant signal obtained.

Also, the authors rely on in-silico prediction algorithms which are mainly built for germline variant interpretation and classification.

Response 2.5.– As indicated above, in the description of the variants selection, we only used the tumor samples to search for variants, discarding the variants that were present in the exomes of the breast tissue control samples. In this way we focus on variants present in the tumors. Furthermore, in the initial variant calling procedure we used the complete database of COSMIC (which corresponds to the *Catalogue of Somatic Mutations in Cancer*), to assign variants. In this way, at the end of all the filters used, we may have some germline variants in our selected datasets, but we gave a clear priority for somatic mutations present in the tumors. These explanations are included in the new manuscript: on **page 3**, **lines 101-103**, on **page 3**, **lines 117-118**, and also on **page 7**, **lines 321-328**.

Allele frequency from ExAC are derived from germline WES analysis. If the authors aimed at exploring the genotype in Colombian women with breast cancer and compare with patients

from other ethnic backgrounds, why not examine germline data? In that way it is possible to exclude somatic and sporadic SNPs.

Response 2.6.– Sorry, but our work does not aim to explore "the genotype in Colombian women with breast cancer and compare with patients from other ethnic backgrounds". The aim of the work is to identify any somatic mutation present in tumors of Colombian women that can be predicted as driver mutation and that is not frequent, or has not been previously described, in Ductal Luminal breast carcinomas (IDC-LM-BRCA), or in other types of cancer. We tried to explain all this in several parts of our new manuscript that have been rewritten. In particular, it can be seen on **page 2**, **lines 79-87**:

> "… the objective of this study was to find a set of gene-centered alterations identified as pathogenic somatic mutations in exomes from invasive breast carcinomas of *Ductal Luminal* subtype, in a cohort of patients of Southwest Colombia; and compare that set with a similar, but larger, cohort of TCGA patients. Pathogenic mutations were detected as somatic tumor variants corresponding to non-synonymous single nucleotide polymorphisms (nsSNPs). The results provide valuable information on the characteristics of this type of breast cancer and allow us to identify novel associations between genetic mutations and related genes in *Ductal Luminal* breast carcinomas. The work revealed well-known *cancer driver* genes, along with some new *cancer driver* mutations associated with the patient subpopulation we studied."

Reviewer 3

Cortes-Urrea et al. clearly present their work based on WES analysis on samples from southwest Colombian women. Thanks to this analysis they confirmed the presence of already reported / known cancer driver mutations, and they described two new driver mutations on CBLB and PRPF8 proteins.

Response 3.1.– Thanks for the general comment and understanding of the work.

Main points:

However, the strength of the take-on message must be revised. In the conclusions the sentence: "Some of the genes (such as ESR1) are very well known and therefore expected, while others (such as CBLB and PRPF8) have been newly detected…" must be rephrased emphasizing the novelty of the mutations detected on CBLB and PRPF8 proteins which have already been associated with breast cancer. [Kang JM, Park S, Kim SJ, et al. CBL enhances breast tumor formation by inhibiting tumor suppressive activity of TGF-β signaling. Oncogene. 2012; 31(50):5123–5131].

Response 3.2.– We thank the reviewer for providing valuable references for our study. We have included the reference by Kang et al. (2012) in the manuscript, and corrected all sentences about CBLB and PRPF8 that referred to these genes as "newly identified" or "newly discovered breast cancer genes", rephrasing to: "genes that present novel mutations". See, for example, in the new **Abstract** and in the **Results**:

On **page 1**, **lines 34-35**:

> "… while others (CBLB, PRPF8) presented driver mutations that had not been reported before. "

On **page 9**, **lines 342-353**:

> "… we found two new driver mutations in two genes that have already been associated with breast cancer: PRPF8 and CBLB. PRPF8 (pre-mRNA processing factor 8) is a component of both U2- and U12-dependent spliceosomes, found to be essential for the catalytic step II in pre-mRNA splicing process. PRPF8 is a cancer related gene with different effect in different tissues and it may affect how RNA binding proteins

mediate cancer-specific phenotypes [40]. CBLB (Cbl proto-oncogene B) encodes an E3 ubiquitin-protein ligase which promotes proteosome-mediated protein degradation by transferring ubiquitin from an E2 ubiquitin-conjugating enzyme to a substrate. It also functions as a negative regulator of T-cell activation. The CBLB gene can block the TGF-Beta pathway and has been associated with breast cancer [41]. In our study, we investigated the relationship between CBLB and the TGF-Beta pathway by analyzing the mutations and the expression levels found for this gene. This is explained in section 3.7."

Furthermore, according to Kang et al. CBLB is overexpressed in the majority of the breast cancer cell lines: a possible explanation of the role of the mutation detected on CBLB protein should be added.

Response 3.3.– As requested by the reviewer, we include a complete new section in the manuscript with a more in-depth analysis of the mutations and alteration of expression found for CBLB gene. This is included on **page 13**, **lines 550-571**:

3.7. Mutations found in CBLB, a gene that inhibits the TGF-Beta pathway

The CBLB gene was one of the two genes for which we identified novel mutations in the Colombian cohort. CBLB and its paralog CBL are called proto-oncogenes and encode E3 ubiquitin-protein ligases. These genes are known to block the TGF-Beta pathway. Indeed, it has been reported that CBL genes enhance breast tumor formation by inhibiting tumor suppressive activity of TGF-Beta signaling pathway [41]. In our analysis of the 476 IDC-LM-BRCA samples from TCGA, the CBL and CBLB genes showed no significant change in expression compared to normal controls. However, two of the genes of the TGF-Beta pathway (TGF-Beta receptors TGFBR2 and TGFBR3) presented a very significant down-regulation in expression (adj.p.value < 0.001 with Limma-Voom and DESEq2), which indicated a probable inhibition of the TGF-Beta signaling pathway in the *Ductal Luminal* breast carcinomas. Furthermore, analysis of the whole exomes of the 476 IDC-LM-BRCA samples from the TCGA project revealed 13 distinct mutations in the CBLB gene, 12 of which had been reported as confirmed somatic tumor mutations (in COSMIC v90), and 6 of them corresponding to missense variants that are likely to damage the protein (**Supplementary Table S5**). Based on these exomes, we also evaluated whether there was any relationship between the mutations and the gene expression of CBLB. Thus, we found in our TCGA set of IDC-LM-BRCA samples, 6 patients with one or more of the 13 CBLB reported mutations. Comparison of the expression of CBLB in these mutated patients with the average expression in the non-mutated patients detected an overexpression of CBLB (adj.p.value = 0.0343) and a repression of a TGF-Beta receptor called TGFBR3L (adj.p.value = 0.0613). Therefore, these 13 mutations may be contributing to a more acute blockage of the TGF-Beta pathway in this group of breast cancer patients. A table with information on these 13 mutations is provided in **Supplementary Table S5**.

Minor points:

An abbreviations list must be added.

Response 3.4.– An abbreviation list has been added at the end to the manuscript on **page 15**, **lines 654-657**.

Some other amendments are indicated in the attached highlighted file.

Response 3.5.– We have done all suggested changes:

(previously: Line 273, page 7): "new genes".
now **page 9**, **lines 342-344**: The sentence has been rephrased
"as shown in **Table 1**, we found two new driver mutations in two genes that have already been associated with breast cancer: PRPF8 and CBLB."

(previously: Line 308, page 8),
Extra space before end point was removed.

(previously: Line 443, page 11),
Extra space in-between “(ER) and” was removed

(previously: Lines 476, 481 and 488, pages 11 and 12),
Ca2+ was replaced by **Ca**

(previously Line 488, page 12),
Na+ was replaced by **Na**
